# pH/redox responsive size‐switchable intelligent nanovehicle for tumor microenvironment targeted DOX release

**DOI:** 10.1038/s41598-023-49446-x

**Published:** 2023-12-18

**Authors:** Fahimeh Badparvar, Ahmad Poursattar Marjani, Roya Salehi, Fatemeh Ramezani

**Affiliations:** 1 Department of Organic Chemistry, Faculty of Chemistry, Urmia University, Urmia, Iran; 2 Drug Applied Research Center and Department of Medical Nanotechnology, Faculty of Advanced Medical Sciences, Tabriz University of Medical Sciences, Tabriz, Iran; 3 Department of Molecular Medicine, Faculty of Advanced Medical Sciences, Tabriz University of Medical Sciences, Tabriz, Iran

**Keywords:** Biochemistry, Chemistry, Materials science, Nanoscience and technology

## Abstract

Tumor microenvironment (TME) targeted strategy could control the drug release in tumor cells more accurately and creates a new opportunity for enhanced site-specific targeted delivery. In this study, (PAA-b-PCL-S-S-PCL-b-PAA) copolymeric nanoparticles (NPs) with size-switchable ability and dual pH/redox-triggered drug release behavior were designed to significantly promote cancer uptake (cell internalization of around 100% at 30 min) and site-specific targeted doxorubicin (DOX) delivery in MDA-MB-231 tumor cells. NPs surface charge was shifted from − 17.8 to − 2.4 and their size shrunk from 170.3 to 93 nm in TME. The cell cycle results showed that DOX-loaded NPs showed G2/M (68%) arrest, while free DOX showed sub-G1 arrest (22%). Apoptosis tests confirmed that the cells treated with DOX-loaded NPs showed a higher amount of apoptosis (71.6%) than the free DOX (49.8%). Western blot and RT-PCR assays revealed that the apoptotic genes and protein levels were significantly upregulated using the DOX-loaded NPs vs. the free DOX (*P*_value_ < 0.001). In conclusion, dual pH/redox-responsive and size-switchable DOX-loaded NPs developed here showed outstanding anti-tumoral features compared with free DOX that might present a prospective platform for tumor site-specific accumulation and drug release that suggest further in vivo research.

## Introduction

Nanotechnology-based therapy has been presented to be a promising approach by which tumoral cells can be treated without damaging healthy cells^1^. Owing to their unique physicochemical and biological characterization, such as the accretion of drug in solid tumors via the retention (EPR) effect with diminished side effects, nanodrug delivery systems (NDDSs) could be considered as one of the most upcoming plans for breast cancer chemotherapy^2^. As of yet, different nanocarriers, including nanogels, liposomes, and polymeric micelles, have been used for anticancer drug delivery^3^. The amphiphilic copolymers with hydrophobic and hydrophilic sections have unique characteristics owing to their distinctive phase activities in the aquatic situation^4^. Copolymeric micelles are provided by the self-assembling amphiphilic copolymers into nano-sized drug delivery systems^5^. Such micelles contain both hydrophobic and hydrophilic characteristics in the core and shell of the structure, respectively^2^. The outcome is the development of unique characteristics systems concerning other nanovehicles: diminutive size-scale leading to the inactive aiming of solid tumors^6^ and also effective cellular internalization^7^, fine solubilization features of hydrophobic blocks incorporated in the lipophilic central section and lengthen blood circulation time generated by the hydrophilic corona blocks from amphiphilic copolymer^8^.

In this study, PCL (poly ε-caprolactone) and PAA (polyacrylic acid) polymers have been considered to prepare amphiphilic copolymers for NPs preparation. Polycaprolactone (PCL) skeleton is a biodegradable polymer certified via FDA^9^. The biodegradation rate of the PCL chain could be enriched by grafting to other water-soluble polymers like PAA, MPEG, etc.^10^. Polyacrylic acid (PAA) is a hydrophilic, non-toxic, biocompatible, and biodegradable polymer that is commonly used in drug delivery, self-assembling and modifying of nanoparticles, and bio-imaging^11^. The carboxyl group of PAA tail increased water-solubility of the polymer and affords modification positions for functionalization and maximizing the loading capacity of anti-cancer drugs^12^. These properties make PCL and PAA good candidates for conventional and novel drug carrier systems^13^. However, inadequate drug release in tumor tissue might hinder the drugs from accessing the aiming site, which impedes their effectiveness in chemotherapy programs. Ideally, the best anticancer drug delivery system needs to have the ability to remain in the bloodstream for substantial time without being eliminated and releasing the drug in the targeted cancer cells^14^.

To satisfy the above concerns, intelligent drug delivery systems have recently attracted tremendous attention^15^. They are categorized into two crucial collections: (i) targeted nano drug delivery systems and (ii) stimuli-responsive nano drug delivery systems^16^. The latter plan could be employed to assist in overcoming the existing challenges in breast cancer treatments due to their controlled—solicitation release of the load in a proper relay to the intracellular microenvironmental conditions, containing temperature^20^, pH^17^, redox^18^, and enzyme^19^, which lead to the high level of anticancer effectiveness with fewer side effects.

Among other things, multi-stimuli responsive nanocarriers, particularly pH/redox dual responsive nanocarriers, have taken particular emphasis and are widely investigated in drug delivery procedures^20^.

According to the conducted studies, the pH range of the tumor microenvironment (TME) is (pH ~ 6.5)^21^. This difference became more dominant in the late lysosomes and endosomes (pH = 4.5–6.5)^22^, compared to extracellular pH in tumor tissues (pH ~ 7.4)^23^. Therefore, pH is the supreme frequently implemented stimulus in tailoring stimuli-responsive nano-drug delivery systems^24^. Charge-shifting NPs illustrated as neutral/negatively charged under normal physiological status can be stimulated to less negative charged (i.e., redox, pH, enzyme, ROS, temperature, or light) to reach extensible blood circulation and improve tumor cellular uptake^25^. Two critical characteristics presented to reach the pH-triggered charge conversion approaches included:

(i) The protonation/deprotonation of surface groups in nanocarriers; (ii) Cleavage of acid-labile bonds, which deprotonation/protonation of polymers present a quicker response to pH changes since no breakage. The protonation/deprotonation of some anionic polymers, etc. happens dynamically exposed to normal physiological pH status^26^. If the pK_a_ range of surface protonatable groups is below physiological pH status, yielding a net negative charge with deportee donated to state and vice versa^27^. Besides, glutathione (GSH) has a high level of intracellular concentration, about 2 − 3 orders more than extracellular plasma^28^.

The stability of disulfide bonds in the structure of nanocarriers in plasma is due to the low concentration of GSH. In contrast, the cleavage of the disulfide bonds of the nanocarrier structure triggered the disintegration of the NPs, which led to the quick release of the cargo, which is caused by the high concentration of GSH^29^.

**Figure 1:**
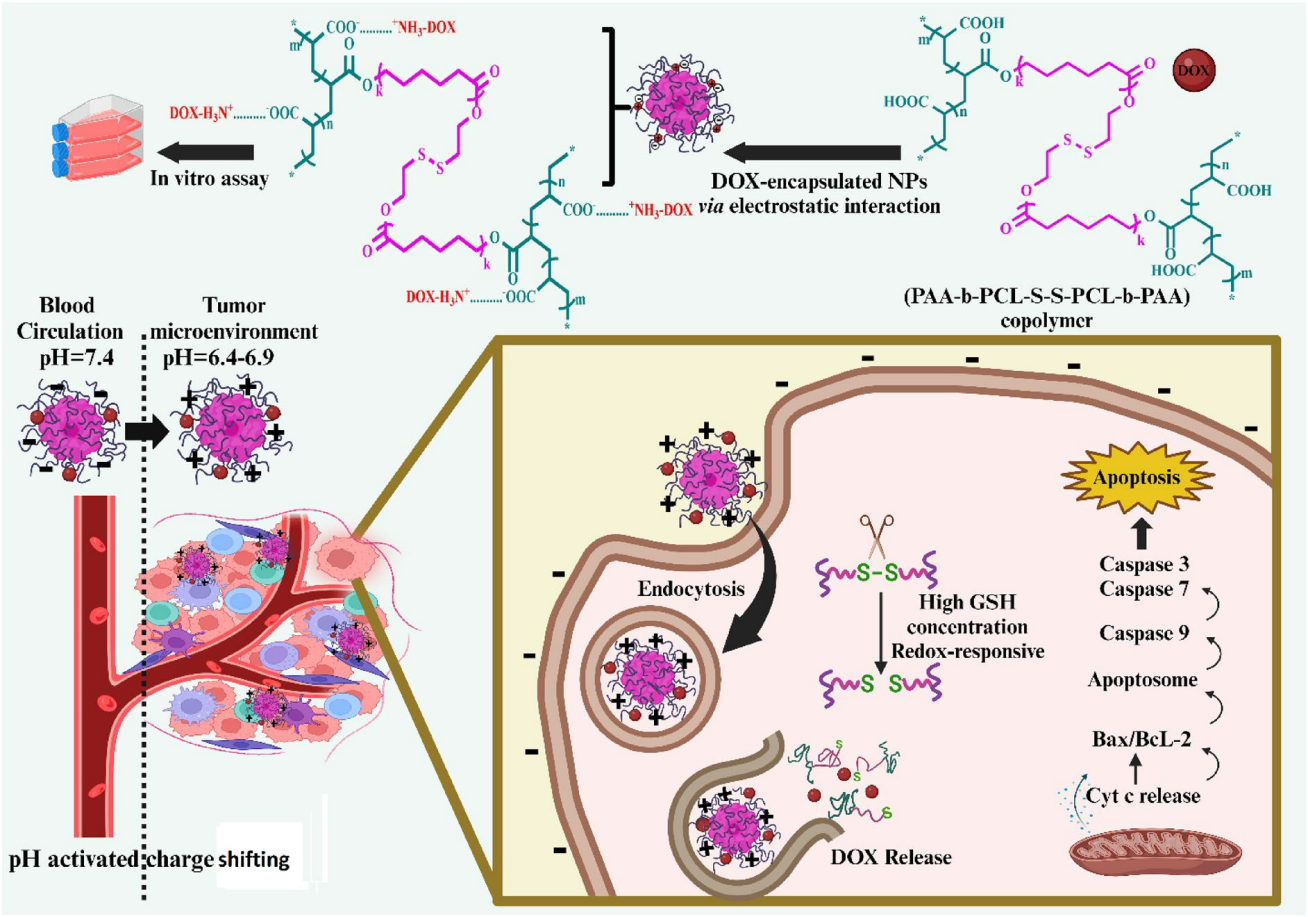
The concept of the research study.

So the significant variance in the GSH concentration and pH outside and inside cells^30^, pH/redox dual responsive nanocarriers might present a high strategic viewpoint for the intracellular release and targeted delivery of therapeutics agents^31^. To achieve this goal, in this study, the smart pH/redox dual-stimuli responsive poly(acrylic acid)-b-poly(ε-caprolactone)-b-poly(acrylic acid) (PAA-b-PCL-S-S-PCL-b-PAA) tri-block copolymeric with size changeable behavior in tumor tissue was designed for on-demand intracellular pH/GSH triggered DOX release. The concept of this research study is shown in Fig. 1.

## Experimental section

### Materials and measurements

The required precursors and reagents purchased for both chemical and biological experiments were as follows: ɛ-Caprolactone (ɛ-CL) (Merck Co), [3-(4,5-dimethylthiazol-2-yl)-2,5-diphenyl tetrazolium bromide)-diphenyltetrazolium bromide] (MMT), Stannous octoate (Alfa Aesar), MCH (2-mercaptoethanol) and NaI (sodium iodide) (Tianjin, China), EtOAc (Ethyl acetate), hydrogen peroxide (30%), Na_2_S_2_O_3_ (sodium hyposulfite), and Na_2_SO_4_ (sodium sulfate) (Beijing Chemical Works Beijing, China), NaOH (sodium hydroxide), (Aldrich), DCC (dicyclohexylcarbodiimide), DMAP (dimethylamino pyridine), PVA (polyvinyl alcohol), DMSO, DMF, methanol, acetone, hexane, diethyl ether (Merck, CO) and PAA (poly acrylic acid, 50 wt% solution in water; ∽ M.W. 5000 (Thermo Scientific Chemicals) was used.

The human breast cancer cell line MDA-MB-231 was supplied by the National cell bank of the Institute Pasteur of Iran. Penicillin/Streptomycin solution 100x (GmbH, Germany), Doxorubicin hydrochloride (DOX-EBEWE Pharma Ges.m.b.H.Nfg.KG A-4866 Unterach, Austria) (Alfa Aesar), Other biological reagents including and rhodamine B (RhB), ribonuclease A, propidium iodide (PI), (Sigma-Aldrich), Furthermore, other substances employed in biological methods such as Trypsin–EDTA, FBS (fetal bovine serum), and RPMI (Roswell Park Memorial Institute) 1640 medium were furnished (Gibco BRL). In all experiments, water (Deionized, resistance > 18.2 MΩ) was utilized. To assess apoptosis, an ApoFlowEx@FITC Kit (EXBIO Praha Inc.) and TRIzol (Life Technologies, USA) were applied. RealQ Plus, SYBR Green Master Mix, 2 × Master Mix Green, and primers were provided from Ampliqon (Denmark) and (Eurofins, Germany), respectively.

### Instrumentation

FT-IR spectra were provided using a spectrophotometer (Nexus-670, Thermo Nicolet, United States). The surface morphology and elemental analysis of the nanoparticles were observed from SEM images obtained by SEM-3200 scanning electron microscope and EDS analysis (MIRA3-FEGSEM, Tescan) technique, respectively. The EE (encapsulation efficiency), release, and DLC (drug loading content) were measured with a UV–Vis spectrophotometer (UV160-Shimadzu, Japan). ^1^H-NMR was conducted on a Bruker DPX-400 NMR spectrometer. Energy dispersive X-ray (EDS) was measured on NumerixDXP-X10P (Carl Zeiss Microscopy GmbH, Germany). Surface charge and particle size of (PAA-b-PCL-S-S-PCL-b-PAA) and DOX-loaded (PAA-b-PCL-S-S-PCL-b-PAA) NPs were evaluated at 25 °C by DLS Zetasizer Nano, Malvern apparatus. Cytation 5 cell imaging multi-mode reader (Bio Tek) was used to determine cell uptake by fluorescent intensity studies, various cell cycles divulge population frequencies, and apoptotic cell analysis. Molecular weight analysis was conducted using gel permeation chromatography Agilent Technologies 1100 Series GPC.

### Synthesis and purification of Bis (2-hydroxyethyl) disulfide (BHES)

For synthesizes of bis (2-hydroxyethyl) disulfide (BHES), 2-mercaptoethanol (MCH) (35.1 mmol, 2.74 g) was dissolved in a stirred solution of EtOAc (50 mL) in a round bottom flask. Then, sodium iodide (0.35 mmol, 52.6 mg) and H_2_O_2_ (30%) (35.1 mmol, 1.93 mL) were added. Afterward, the obtained mixture was stirred (30 min) before adding saturated aqueous Na_2_S_2_O_3_ (15 mL). The resulting mixture was extracted with AcOEt (3 × 16 mL). The solution was dried, and the solvent was evaporated. The residue was purified by silica gel column chromatography (EtOAc and n-hexane) to afford the pure products (2.61 g, 95%). ^1^H-NMR and FT-IR spectroscopy were used for structural evolution.

### Synthesis and purification of HO-PCL-SS-PCL-OH

The ROP (ring-opening polymerization) process of ε-CL using HES as an initiator with the help of a catalyst (stannous octoate) formed HO-PCL-SS-PCL-OH. Generally, under an argon atmosphere, HES (1 mmol, 154 mg), stannous octoate (0.172 mmol, 70 mg), and ε-CL (37.5 mmol, 4.28 g) were added into a dried Schlenk flask. Four freeze–pump–thaw cycles deoxygenate the final mixture under magnetic stirring at 100 °C for 20 h. After 30 min, the polymerization process was done by placing the flask into liquid N_2_. After cooling the crude product to room temperature, it dissolved in dichloromethane. HO-PCL-SS-PCL-OH was gained by precipitating from Et_2_O and, after that, precipitated in MeOH and dried. The structure of the product was evaluated by FT-IR and ^1^H-NMR spectroscopy. GPC was utilized to evaluate the weight of molecular (M_w_) and dispersity (Đ).

### Synthesis of polyacrylic acid-b-poly(ε-caprolactone)-b-polyacrylic acid PAA-b-PCL-S-S-PCL-b-PAA triblock terpolymer

The polyacrylic acid-b-poly(ε-caprolactone)-b-polyacrylic acid triblock terpolymer formation was performed by the Steglich esterification method with dicyclohexylcarbodiimide as a coupling reagent and 4-dimethylamino pyridine as a catalyst.

At first, DCC (0.41 mmol, 84 mg) and DMAP (0.048 mmol, 5 mg) were dissolved in a 50 mL anhydrous DMF in a tri-necked round bottom flask equipped with a gas inlet and outlet, dropping funnel, and magnetic stirrer. Then, it was transferred into the ice bath. The mixture was degassed through argon bubbling (10 min). Next, the excess amount of dissolved poly acrylic acid (0.5 mmol, 4 g) in the anhydrous DMF was added quickly into the reaction mixture under an inert atmosphere and degassed through argon bubbling for 15 min. In the third step, a defined amount of HO-PCL-SS-PCL-OH (0.17 mmol, 1 g), which had already been wholly dissolved in anhydrous DMF, was added dropwise under argon gas under a funnel for half an hour. After 30 min of stirring at 0 °C, the temperature of the reaction was up to 25 °C and stirred for two days. Finally, the expected target copolymer (PAA-b-PCL-b-PAA) white powder can be separated from the excess unreacted polyacrylic acid by adding a large amount of Et_2_O and then precipitated in cold methanol and lyophilized. The PAA-b-PCL-ss-PCL-b-PAA terpolymers were characterized by ^1^H-NMR and FT-IR.

### Nanoparticles preparation and characterization

The copolymer developed here self-assembled into NPs by the following method, presenting an external hydrophilic shell and an internal lipophilic core.

To prepare a blank polymeric NPS, firstly, freeze-dried and synthesized copolymer (100 mg) was dissolved in DMSO (3 mL) with the dropwise addition of the polymer solution to a cooled PVA (25% w/v solution, 20 mL) while agitated via sonication probe. Then the solution of the blank micelle was transferred into Amicon® centrifugal filters to be centrifuged at 4500 rpm (10 min). The dialysis membrane product was collected and lyophilized for further usage.

Afterward, doxorubicin (10 mg) was dissolved in PVA (0.25% w/v, 20 mL) to prepare the drug-loaded polymeric NPs. The weight ratio of the copolymer to the drug was 10 to 1, and drug loading was actualized at pH = 7.4. Next, a polymer solution containing 100 mg in 3 mL DMSO was added into the PVA under a sonication probe in an ice bath, after that the DOX–PVA pH was adjusted at 7.4 with sodium hydroxide. In the end, the DOX-loaded NPs were centrifuged at 4500 rpm (10 min). The obtained product was lyophilized and kept at – 20 °C.

The zeta potential and diameter of the drug-loaded and blank NPs in the aqueous solution were investigated by DLS analysis^11^. Also, the morphology, size distribution, and elemental chemical composition of the NPs were characterized by SEM and EDS analysis^12^.

The colloidal stability of NPs in deionized water and PBS buffer was assessed by DLS data.

To realize the charge-shifting ability of nanoparticles surface in PBS at pH = 7.4 (physiological conditions agents) to pH = 6.5 (tumor extracellular), zeta analyses were conducted after 4 h incubation.

The EE of NPs was assessed in micellar solution (0.1 mg/mL) on a UV–Vis spectrophotometer by checking the absorbance at 480 nm. EE was evaluated as the equation^13^:

$$ {\text{DEE}}\,\left( \% \right) = \frac{{\text{Mass of drug in nanocarrier}}}{{\text{Mass of feed drug}}} = 100 $$

### GSH-triggered change of the nanoparticle sizes

The disulfide bonds as stimulus-sensitive functionalities within the micelle network change the size of the nanoparticles due to their cleavage using high concentrations of reducing agents, including decreased glutathione (GSH), thioredoxin, and peroxiredoxin, which are found at various levels of all over the body^32^. GSH as a reducing agent was selected in our degradation experiments^33^. The size change of reduction breakable NPs in response to GSH (10 mM) in PBS buffer (pH = 6.5) was assayed with DLS^34^. The nanoparticle samples (10 mg) were dispersed in PBS (10 mM GSH, pH = 6.5) respectively and then divided into four aliquots. Afterward, one component was considered for control, and the rest were modified by adding GSH. All aliquots were incubated under their conditions (0 h without GSH addition, 0.5, 3 h, and 1 day with the addition of GSH (10 mM), and all at pH = 6.5), and then DLS studies were performed.

### In-vitro pH and GSH dual-triggered intracellular release of doxorubicin

The pH and redox effect of cumulative doxorubicin release profiles from the pH/redox dual stimuli-nanosystem were further investigated in the following way^35^.

Drug-loaded NPs (5 mg) were suspended in a sink solution (2 mL) with four different values of release media (pH = 6.5, pH = 6.5 + 10 mM GSH, pH = 7.4, and pH = 7.4 + 10 μM GSH). Next, the samples were situated in a shaker incubator during the release study at 40 °C (for pH = 6.5) and 37 °C (for pH = 7.4). After predetermined times (1, 2, 3, 4, 24, 48, 72, 96, 120, and 144 h), the samples were centrifuged (25 min, 8000 rpm), and the solution of supernatant was assembled and substituted with new sink solution in the same amount. After suitable dilutions, the samples were quantified using UV absorptions at λ_max_ = 233.5 nm. The percentage of doxorubicin release was estimated using the following equation:

$$ {\text{Drug release}}\,\left( \% \right) = \frac{{\mathop \sum \nolimits_{t}^{0} \left( {{\text{Amount of drug in release medium at time}} t} \right) }}{{\text{Amount of drug loaded in nanocarrier}}} \times 100 $$

### Statistical analyses

The experiment results were performed in triplicate for all operating variables studied. Results are articulated as the mean value, and the within-process standard deviation is calculated for each set using GraphPad Prism software (v. 9) or Excel. If the *P*_value_ is 0.05 or lower, the results were considered statistically remarkable.

### Cell culture

In streptomycin (50 mg/mL), penicillin (1%, 50 IUmL^−1^), RPMI-1640 medium (10%, FBS), and a specified incubator with CO_2_ (5%) at 37 °C, the MDA-MB-231 cancer cell line of the breast (Institute Pasteur-Iran) was cultured.

### Cytotoxicity of synthesized PAA-b-PCL-S-S-PCL-b-PAA-NPs in MDA-MB-231 cancer cell line breast

The cytotoxicity profile of DOX-loaded (PAA-b-PCL-S-S-PCL-b-PAA) copolymeric NPs against the MDA-MB-231 breast cancer cell line was surveyed through an MTT assay. While the cell residents reached 70% confluence, cells were separated with trypsin (0.25%) in PBS and centrifuged (5 min, 2500 rpm). After that, to attach the cells to the bottom of the well, cells with a density of 5 × 10^3^ (cells/well) were seeded in 96-well microplates in 200 μL RPMI 10% FBS and further incubated at 37 °C for one day.

Then, cells were treated with eight subsequent dilutions of the drugs in triplicate equivalent concentrations (0.156–10 μg·mL^−1^) of DOX-loaded NPs. DOX was provided in a new cell growth medium with FBS (2%) and incubated in continued for two days. Cells without pretreatment were employed as the control. After rinsing the removed medium with PBS, the RPMI (150 μL), FBS (2%), and MTT (50 μL of 5 mg/mL) assay stock solution in PBS was added and incubated (4 h). The unreacted medium with MMT was separated. The attained blue formazan crystals were dissolved in DMSO, and after shaking (10 min), the absorbance was evaluated (570 nm) using a microplate ELISA reader. The experiment was repeated. Statistical analyses of all tests were performed. The half-maximal IC_50_ (inhibitory concentrations) values were estimated based on the percentage of treatment over control.

### Intracellular uptake study

The qualitative and quantitative uptake of rhodamineB-labelled blank and DOX-loaded NPs by the MDA-MB-231 cell line has been carried out by cytation five cell imaging multi-mode reader (Bio Tek) and FACScalibur flow cytometer, respectively.

rhodamineB-labeled NPs were reached in the following way: first, a copolymer (10 mg) was added into DMSO (1 mL) and stirred (several hours). Next, the result from the solution was added drop wisely into PVA solution (4 mL, 0.25% w/t) was mixed with sonication in the dark condition at reduced temperatures while having 50 μL of 0.1 mg/mL rhodamine-B (RhB). In continuation, the RhB-labelled NPs were collected by Amicon® and rinsed several times to remove the unloaded RhB dye. Finally, the RhB-NPs were dispersed in water (1 mL) and frozen for future utilization.

The MDA-MB-231 were seeded in 6 well plates at a density of 5 × 10^5^ cells/well and incubated (2 days). Cells (70% confluence) were treated with DOX-loaded NPs and RhB-labelled NPs, while the untreated ones acted as the negative control. After incubation (0.5, 1.5, and 3 h), PBS was utilized to wash the cells, then trypsinized and harvested. Rhb-labeled NPs uptake was counted up using a FACScalibur flow cytometer. The uptake of the RhB-labelled NPs at the culture media, adjusted to 7.4 or 6.5 to simulate the physiological condition or TME.

The intracellular uptake of the NPs was confirmed by cytation five cell imaging multi-mode reader. The MDA-MB-231 has been grown according to a quantitative method, and after incubation with RhB-labelled NPs and DOX-loaded NPs (0.5, 1.5, and 3 h), the cells were rinsed with PBS, and RhB-labelled nanocarrier uptake was visualized and photographed.

### Cell death mode: apoptosis detection by flowcytometric method

PI staining (Annexin V/PI) and Annexin V-FITC have been employed for evaluating the percentage of dead cells by apoptosis and necrotic pathway. Thus, we determined the mechanism of cell death using an Ex-bioscience apoptosis kit. The cultivation of MDA-MB-231 cells (6-well plate, 5 × 10^5^ cell/well) was accomplished and incubated (two days, 5% CO_2_, at 37 °C), followed by treatment with a concentration at the IC_50_-value of all samples (free drug, blank NPs, drug-loaded NPs). After incubation (4 h), the medium was refreshed by changing it with the new one after the removal of the remaining NPs and drugs and returned to the incubator (two days). Briefly, PBS was used to rinse the collected supernatants and poured them into the related tubes. Thereafter, cells were trypsinized, rinsed with PBS, centrifuged, and their supernatants were eliminated without further use. As reported by the manufacturer's process of the Annexin V staining kit, the cells were rinsed with an annexin binding buffer, consequently, resuspended in binding buffer again and next, in Annexin V-FITC (5 mL) after that, propidium iodide (5 mL) was added to all the samples and mixed gently. Incubation is continued for 15 min at room temperature. Immediately, the cells were centrifuged, and the pellet was re-suspended in binding buffer (100 mL). Untreated cells were considered negative controls, while treated cells with blank NPs were recognized as positive controls. All analyses were executed in triplicate, and obtaining results were depicted as mean values of Annexin V kit parameters ± SD (standard deviation). As a final point, the samples were instantly checked via a FACSCalibur flow cytometer. The unstained sample was also analyzed as the auto-fluorescence reference.

### Cell cycle arrested analysis

The cell cycle arrest effect of free drug, blank-NPs, and DOX-loaded NPs was evaluated using a Cell Cycle assay. MDA-MB-231 cell seeding was done in a 6-well culture plate (5 × 10^5^ per well), and attachment was conducted (2 days). The cells were treated at IC_50_ drug dosage with DOX-loaded NPs and DOX. After incubation (4 h), the medium was refreshed with the new one and incubated (two days) again. Non-treated cells were set as the negative control and blank NPs as the positive control. Afterward, the cells were rinsed with a completely fresh medium to remove the remaining NPs and drugs. After that, the cells are trypsinized and harvested into their tubes. Cells in tubes were centrifuged immediately; then, their supernatant was discarded and resuspended pellet in fresh and cold PBS (700 µL) and centrifuged again. Carefully aspirated supernatant, PBS (300 µL) poured into every cell-containing tube and resuspended cells by gentle vortexing. At last, the fixation of cells was done using EtOH and incubated (at 4 °C for three days). Then, all tubes were rinsed and centrifuged several times, and their supernatants were replaced with PBS (300 µL), and later, ribonuclease A (10 µL) replenished the tubes. The cells were stained by propidium iodide (PI) after incubation (45 min) and kept in a dark place (10 min). Finally, the population profile detection of cell cycle phases was estimated with a FACSCalibur (Becton Dickinson).

### RNA extraction and cDNA synthesis and quantitative PCR

MDA-MB-231 cells were seeded in a density of 106 cells per well in 6 well plates. After one day, the cells were treated by the blank micelle, DOX, and DOX-loaded NPs at their IC_50_ provided by MTT assay and left for 4 h. After this time, the culture media was refreshed with the new one and incubated (two days).

Cells treated with blank micelle were considered as the positive control. Also, none treated cells were considered as the negative control. Total RNA was secluded by the TRIzol method from MDA-MB-231 cell lines. Concisely, using a centrifuge, the precipitated are obtained at 500 g at 4 °C. The cell lysis occurred by adding TRIzol™ (750 μl) reagent and CHCl_3_ (200 μl), respectively. Then securely caped the tubes and incubated for 2 min at room temperature.

The samples were centrifuged (at 4 °C, 12,000 g for 20 min) and a phase of aqueous was assembled, and 2-propanol (1 mL) was poured into sedimenting the total RNA at 12,000 g (20 min, at 4 °C). To rinse the RNA plate, ethanol (75%) was added, and the total RNA was dissolved in DEPC-treated water (20 λ). The cDNA creation was done through Revert Aid Reverse Transcriptase according to the manufacturer protocol exactly. The reactions were triplicated using glyceraldehyde-3-phosphate dehydrogenase (GAPDH) as a reference gene to record the Ct (threshold cycle) value.

The widely accepted comparative “ Ct “ procedure (also known as the ΔΔCt procedure) was employed to analyze real-time PCR data. The primers are mentioned in Table S1. The real-time qPCR (quantitative PCR) technique is frequently utilized to detect the apoptosis routes in the following PCR: preliminary denaturation (15 min at 95 °C), denaturation (15 s at 95 °C), and annealing/extension (50 s at 60 °C). The qPCR mixture including cDNA (2 μl), 2 × SYBR Green Master Mix (5 μl), primer pair mix (0.5 μl of 5 pmol/μl), and H_2_O (3 μl). GAPDH and –ΔΔCt were employed as the housekeeping gene and evaluated the fold alterations.

### Cell lysis and western blotting

For western blotting, MDA-MB-231 was seeded in 6 well plates (106 cells/well) and incubated (1 day). Cells were treated by PAA-b-PCL-S-S-PCL-b-PAA copolymeric micelle at IC50 value acquired by MTT assay and then incubated (2 days). Next, cell tissues were homogenized with cold bath therapy via RIPA lysis buffer containing protease inhibitor cocktail one tablet, EDTA (3 mg), Tris–HCl (500 μL, pH = 8), NaCl (80 mg), Sodium deoxycholate (25 mg), SDS (10 mg) and Triton [(% NP40 (1%), 10 μl]. The supernatant was gathered through s centrifuge (1200 RPM, 40 °C, 20 min). Protein quantification concentration was determined by Bradford assay with w1-8itc commercial kits and evaluated via the spectrophotometric instrument. Protein samples were separated by sodium dodecyl sulfate–polyacrylamide gel electrophoresis containing protein/well (10 μg). The target bands were transferred onto the blotting holder with a membrane of PVDF (polyvinylidene difluoride). Unspecific bands were masked by employing a blocking buffer containing skim milk [5% (w/v)] in Tween®20 (TBST, 1 × TBS 1%), next, incubated overnight at 4 °C with specific primary antibodies with a ratio of 1:1000 are caspase 3 cell signal rabbit MAb, caspase 7 (C7, cell signaling) rabbit polyclonal antibodies (PAbs), Bax, mouse monoclonal antibody (Mab), caspase 9 (cell signaling) rabbit Pabs, GAPDH mouse MAb, Bcl2 sc-92 Mab, which added to PVDF membrane.

Incubated the secondary antibodies (caspases 3, 7, 9 and mouse anti-rabbit IG-HRP, and m-IgGκ BP-HRP were utilized for GAPDH, Bax (B9), and Bcl-2 in TBST with milk (5%) for 1 h, and subsequently wash membrane three times with TBST for 5 min to remove residual TWEEN. The membranes with secondary antibodies diluted 1:1000 in the blocking buffer were incubated (1 h). A professional chemiluminescence detection kit was used for the visualization of blots. For study and check the capture and analysis of the high-resolution digital images, an Amersham Imager 600 was used for all measurements. By application of Image J software 1.52n in bands quantifying, mentioned bonds were quantified, and as a loading control, the GAPDH was employed.

## Results and discussions

The synthesis root of PAA-b-PCL-S-S-PCL-b-PAA triblock copolymer were shown in Figure S1.

### Characterization

#### ^1^H-NMR, FT-IR, and GPC analysis

FT-IR analysis: The FT-IR spectra of the HS-CH_2_CH_2_–OH (Fig. S2A), HO–(CH_2_)_2_-SS-(CH_2_)_2_–OH (Fig. S2B), HO-PCL-SS-PCL-OH (Fig. S2C), PAA (Fig. S2D), and (PAA-b-PCL-S-S-PCL-b-PAA) (Fig. S2E) associated with detailed explanation were presented in supplementary file.

GPC study: The GPC diagram of HO-PCL-SS-PCL-OH polymer is presented in Fig. S3 in the supplementary file. The precise description of the GPC study was presented in supplementary file.

**Figure 2:**
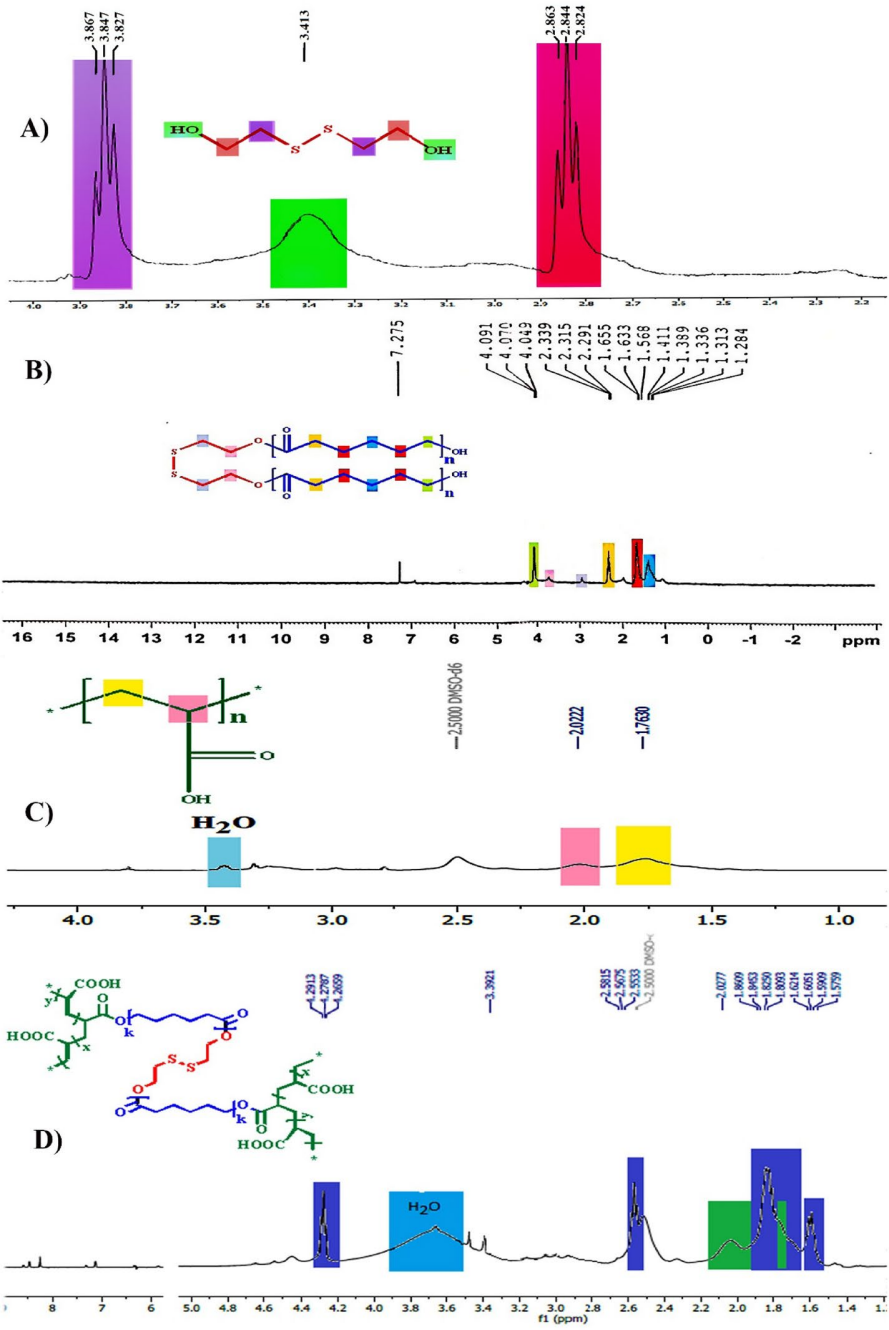
^1^H-NMR spectra of HO–CH_2_CH_2_–SS–CH_2_CH_2_–OH (BHES) in CDCl_3_ (**A**), HO-PCL-SS-PCL-OH in CDCl_3_ (**B**), polyacrylic acid in DMSO-*d*_*6*_ (**C**), and PAA-b-PCL-S-S-PCL-b-PAA copolymers in DMSO-*d*_*6*_ (**D**).

^1^H-NMR analysis: The structure of polymer synthesis steps was estimated with ^1^H-NMR. The detailed ^1^H-NMR related to the products of every stage have appeared in the spectrum, including HO–CH_2_CH_2_-SS-CH_2_CH_2_–OH (BHES) in CDCl_3_ (Fig. 2A), HO-PCl-SS-PCl-OH in CDCl_3_ (Fig. 2B), polyacrylic acid in DMSO-*d*_*6*_ (Fig. 2C) and (PAA-b-PCL-S-S-PCL-b-PAA) in DMSO-*d*_*6*_ (Fig. 2D).

According to Fig. 2A, peaks related to BHES in δ (ppm): 2.84 (t, HOCH_2_**CH**_**2**_S-S**CH**_**2**_CH_2_OH), 3.41 (s, **H**OCH_2_CH_2_S-SCH_2_CH_2_O**H**), 3.84 (t, HO**CH**_**2**_CH_2_S-SCH_2_**CH**_**2**_OH), confirmed the correctness of the BHES structure.

Figure 2B shows characteristic peaks of the HO-PCL-SS-PCL-OH in δ (ppm): 1.28–1.41 (m, –OCH_2_CH_2_**CH**_**2**_CH_2_CH_2_CO–), 1.56–1.65 (m, –OCH_2_**CH**_**2**_CH_2_**CH**_**2**_CH_2_), 2.31 (t, –OCH_2_CH_2_CH_2_CH_2_**CH**_**2**_CO–), 3.72 (t, –O–**CH**_**2**_CH_2_-S-S-CH_2_**CH**_**2**_–O–), 4.07 (t, –**CH**_**2**_OH), 4.33 (t, –O–CH_2_**CH**_**2**_-S-S-**CH**_**2**_CH_*2*_–O–).

As shown in Fig. 2C, related to polyacrylic acid, the characteristic peaks in δ (ppm): 1.76 (–**CH**_2_) and 2.02 (–CH_2_CH_2_–**CH**COOH) are observed.

Figure 2D shows the peaks of PAA-b-PCL-S-S-PCL-b-PAA in δ (ppm): 2.02 is associated with the PAA backbone protons. A shoulder of methylene peaks of the PAA overlapping with methylene peaks of the PCL backbone can be seen in region 1.70 ppm. All peaks of the PCL backbone was observed as follow: 1.57–1.62 (m, –O(CH_2_)_2_**CH**_**2**_(CH_2_)_2_CO–), 1.80–1.87 (m, -OCH_2_**CH**_**2**_CH_2_**CH**_**2**_CH_2_), 2.56 (t, –O(CH_2_)_4_**CH**_**2**_CO–), 4.27 (t, –**CH**_**2**_OH). Peaks shift are noticeable in the PCL backbone.

#### Morphology

**Figure 3:**
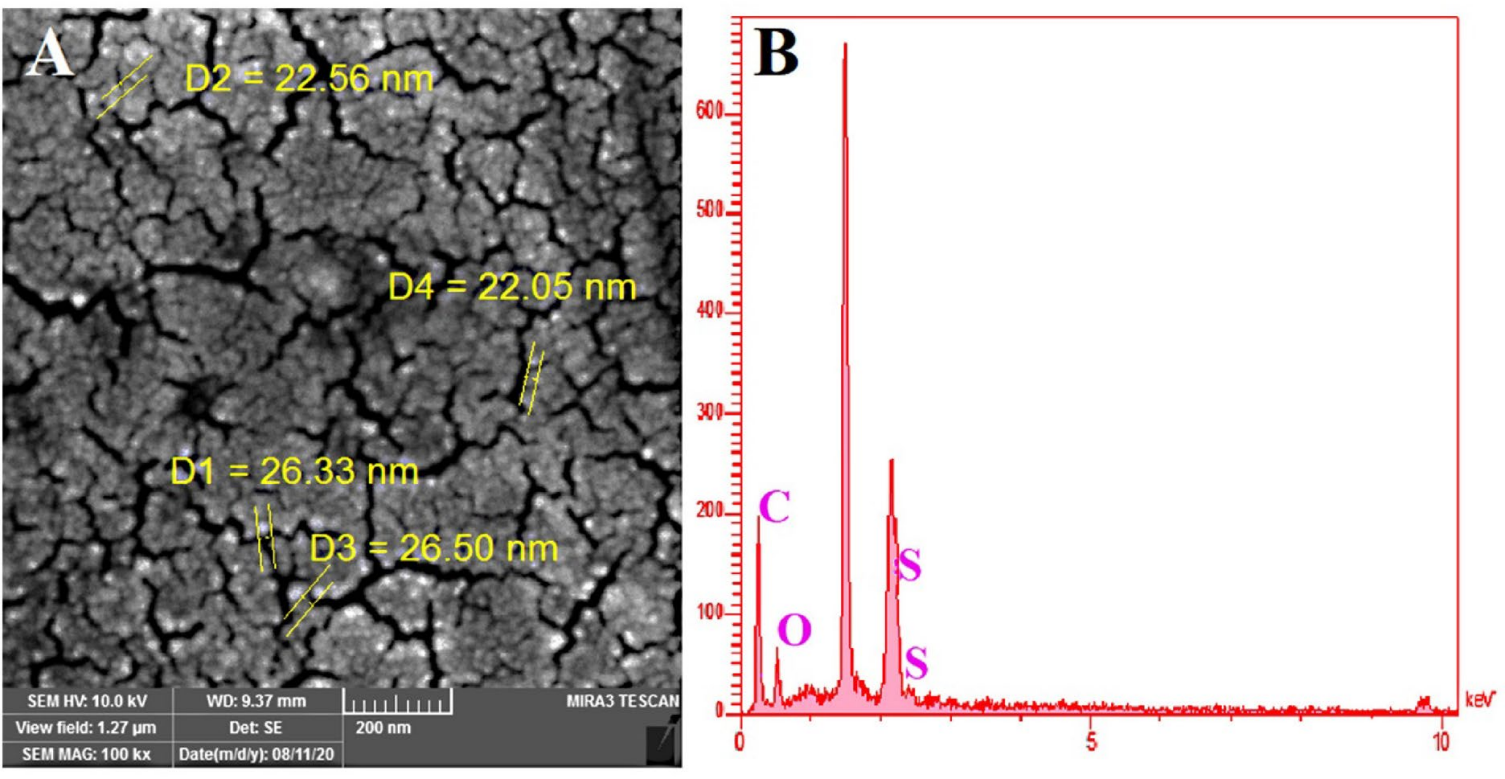
SEM image (**A**) and EDS analysis of PAA-b-PCL-S-S-PCL-b-PAA blank NPs (**B**).

EDS and SEM are widely employed to characterize the size and surface morphology of the NPs. The SEM images of desirable NPs are displayed in Fig. 3A. These images revealed that the NPs have a spherical structure and regular morphology with an average diameter of 25 nm.

EDS is an X-ray spectroscopic technique used to identify the elemental composition of materials, which is performed along with the SEM analysis. The EDS data illustrated in Fig. 3B. demonstrated the presence of carbon, oxygen, and sulfur elements. The presence of the S in the final nanocarrier is evidence of disulfide bond formation within the NPs network.

#### Nanoparticle characterization and pH-triggered charge-shifting behavior of engineered nanoparticles

Size distribution, and surface charge of (PAA-b-PCL-S-S-PCL-b-PAA) NPs and drug-loaded NPs were featured by the DLS-Zeta technique.

In Figure S4A, the size of NPs obtained a monomodal distribution with hydrodynamic diameters around 170.3 nm, while after drug loading, it increased to 277.4 nm (Fig. S4B). The diversity of the nanoparticles’ size (reached from DLS and SEM measurements) can be described due to the hydration layer stuck to its surface during the execution of the size distribution by the DLS technique. In contrast, SEM on freeze-dried demonstrated an approximately slender apportionment of virtually spherical particles^36^.

The surface charge of free NPs presented a negative value of − 17.8 mV (Fig. S4A), and the surface charge of drug loaded-NPs reached + 6.8 mV, as can be seen in Figure S4B. These changes confirmed the loading of DOX during the NPs preparation.

The colloidal stability of NPs after 2 mounts in PBS and distilled water was confirmed by DLS technique. Particle size in PBS and distilled water obtained 323 (Fig. S5A) and 578 nm (Fig. S5B) and PdI was 0.414 and 0.335, respectively.

Stabilization of synthesized (PAA-b-PCL-S-S-PCL-b-PAA) polymeric NPs is associated with their anionic polyelectrolyte polyacrylic acid (PAA) arms suggesting electrostatic repulsion as stabilization main mechanism. Moreover, hydrogen-bonding helps to more stability of NPs. The colloidal stability of PAA-based NPs was previosely reported in literature^37–39^.

The lower pH = 6.5 in tumor tissues compared to pH = 7.4 in normal tissues is due to the current high rate of glycolysis in cancer cells^40^. By considering this unique behavior of tumor tissue, pH-sensitive NDDSs are designed for the safe transportation of drugs in the physiological pH environment and release the drug smartly when the pH triggering point is reached in tumor tissue^22^. PH of the late endosomes and lysosomes has been estimated to be at much more acidic pH (in the region of 4.5–5.5). Some nanocarriers are absorbed via assimilation and endocytosis within lysosomes and endosomes^41^. So, this pH difference is noteworthy for pH-sensitive nanocarriers. The charge-shifting capability of designed NPs in this study was assessed with an investigation of charge shifting of NPs surface at pH = 7.4 (normal tissues) and pH = 6.5(TME)^42^. According to Fig. S6 the zeta potentials switched sharply from − 17.8 at PBS buffer with pH = 7.4 to − 2.44 mV at PBS buffer with pH = 6.5 after a 4 h incubation, respectively. It should be attributed to the protonation of carboxylate groups, and the osmotic pressure of counterions^43,44^.

#### Reduction-induced size shrinkage of the nanoparticles under in vitro reducing intracellular condition of tumor tissue (GSH = 10 mM and pH = 6.5)

**Figure 4:**
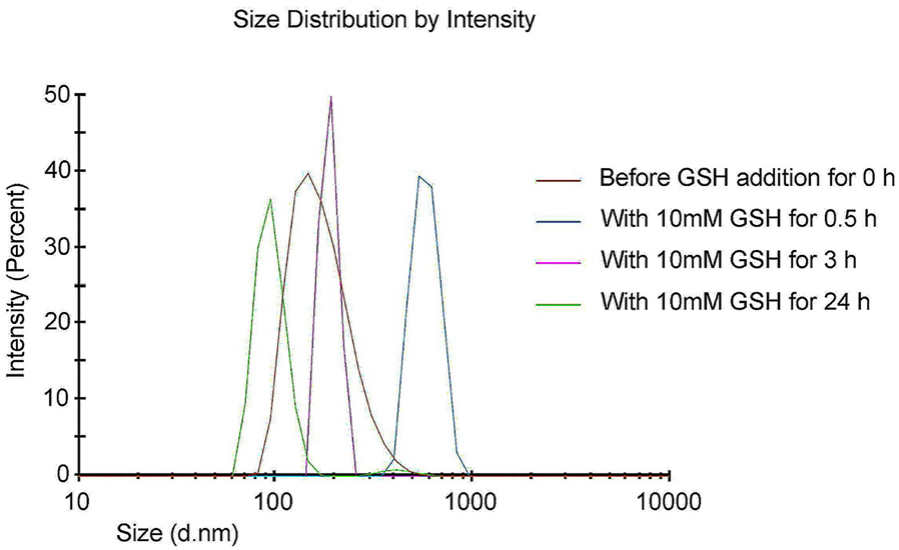
Size distribution of nanoparticles under in vitro reducing environment of intracellular condition with 10 mM of GSH (pH = 6.5).

To check GSH triggered size change of our developed NPs, the size change of PAA-b-PCL-S-S-PCL-b-PAA nanoparticles upon exposure to pH = 6.5 and 10 mM GSH concentrations in definite time intervals (0.5, 3, and 24 h) was monitored using DLS measurement. As depicted in Fig. 4, the particle size of NPs before the GSH addition was 186.7 nm. When the GSH concentration in the medium was coped with 10 mM, accelerated decrease in size was achieved. In the first 0.5 h, total NP’s size reaches over 560.3 nm. A more significant size change was detected after 3 h (489.9 nm). It reaches a diameter under 93 nm after 24 h. The shrunk nanoparticles' size depends on the cleavage of the disulfide linkages in the structure of the copolymeric NPs. The millimolar density of GSH can separate the disulfide bonds by oxidation–reduction reaction^45^. Similar study was established in Guo’s article, in which disulfide bonds corporate in PEG-PLA-S-S PEI-DMA NP’s structure were cleaved. Consequently, the remarkable size shrinkage permitted DOX release for DNA interruption^46^.

#### Entrapment efficiency study

The encapsulation efficiency of DOX-loaded (PAA-b-PCL-s-s-PCL-b-PAA) NPs was obtained at 93.54%. The reason behind high drug encapsulation efficiency was the presence of electrostatic interaction between the positively charged -NH_2_ section of DOX at physiological conditions^47^ and negatively charged –COO^−^ sections of the polyacrylic acid tail of unique structure of the NPs.

#### In vitro pH/redox triggered DOX release study

To explore whether nanoparticles represented pH/redox dual-responsive drug release pattern, DOX release assay was carried out by suspending 1 mg of DOX-loaded NPs in different release media including: (i) in PBS (pH = 6.5), (ii) in PBS (pH = 6.5 and 10 mM GSH), (iii) in PBS (pH = 7.4 and 10 μM GSH), and iv) in PBS (pH = 7.4) all at 37 °C. Fig. S7 represented the pH and GSH dependence of DOX release from the copolymeric NP solutions. As expected, the release rate was relatively low in PBS plus GSH (10 µM) and reached 29%, and it is even lower for PBS without GSH (7%) after one day. In contrast, redox/pH dual-responsive DOX release reached as high as 91% within the first 24 h, at pH = 6.5 plus GSH 10 mM, which was remarkably higher than other mediums and revealed the accomplished DOX release from the NPs in the cancer intracellular milieu. This fast cumulative release of DOX in 10 mM GSH medium was likely because NPs following degradation were destabilized and formed drug diffusion channels. These NPs also showed pH-responsive DOX release, and additive release at pH = 6.5 was dramatically enhanced compared to that at pH = 7.4 in the presence or absence of equal GSH concentration, which indicates the rapid release of DOX from NPs in TME. One possibility is due to the protonation of PAA carboxylic groups at pH = 6.5 which leads to the removal of ionic interaction between DOX amine groups and PAA carboxylic groups, which decreases the size of the NPs at pH = 6.5. It causes the discharge of therapeutics from the NPs and, afterward the enriched drug release. Moreover, DOX solubility was promoted at pH = 6.5, which might enhance the drug release. These approaches showed that the highest DOX release was achieved in pH = 6.5 and GSH 10 mM due to the protonation of carboxyl units in the shell of the NPs^48^ and disruption of the nanoparticle core due to the splitting of disulfide bonds in the biodegradable disulfide bond-bridged copolymer under reducing environment of intracellular (GSH = 10 mM) (Fig. S8)^49^. Li et al. investigated a study about Stimuli-responsive clustered nanoparticles for improved tumor penetration and therapeutic efficacy, that nanoparticles after reaching the TME, were able to respond to extracellular acidity and release their cargo^50^. Kim et al. synthesized DOX-loaded pH-sensitive poly (ethylene oxide)-b-poly (methacrylic acid) NPs via electrostatic interactions. The hydrophobic interactions between the ammonium groups in the daunosamine moiety and anthracycline residues of the DOX molecule afford stabilization of the complex. Throughout the first hour at pH = 6.5, up to 50% of the DOX was released because of the protonation of carboxylic groups in the core of the NPs^51^.

Based on these achievements, these pH/redox-sensitive biodegradable NPs with triggered drug release behaviors are incredibly motivating as “smart” vehicles. They seem a good approach to launch successful payload release under tumor-relevant conditions.

#### Study of cell viability following therapy of MDA-MB-231 cells with DOX-loaded PAA-b-PCL-S-S-PCL-b-PAA

To evaluate the IC_50_ value of the DOX-loaded (PAA-b-PCL-S-S-PCL-b-PAA) and DOX alone, MDA-MB-231 cell lines were exposed to increasing concentrations of the drugs for 48 h. The DOX-loaded NPs showed significantly higher toxicity (IC_50_ = 0.386 µg·mL^−1^) against MDA-MB-231 cells compared to DOX alone (IC_50_ = 0.607 µg·mL^−1^) (Fig. S9). Poursalehi et al. study demonstrated that P(NIPAAm-MA-g-CA) super acid polymer coating reduced the toxicity of gold nanoparticles (SAP@TBP@AuNPs nanocomposite) and also, prepared an excellent site for pH-interactive ionic interaction with DOX because of high quantity of carboxylic acid groups on the polymer surface. Rahmani et al. synthesized P(CA-g-PMA-co-PLGA (citric acid-grafted poly maleate-co-poly lactic-glycolic acid) for DOX delivery to MDA-MB-231, and the results showed that toxicity of DOX loaded NPs (IC_50_ = 0.157 µg·mL^−1^) was higher than DOX (IC_50_ = 0.569 µg·mL^−1^)^52^.

#### Investigation of intracellular uptake of DOX-loaded (PAA-b-PCL-S-S-PCL-b-PAA) by MDA-MB-231 cell line

To investigate the cellular uptake in a time-dependently manner, DOX-loaded (PAA-b-PCL-S-S-PCL-b-PAA) NPs and blank NPs were labeled with rhodamine B (RhB), and the internalization process of RhB-containing nanoparticles on the MDA-MB-231 was evaluated (0.5, 1.5 and 3 h of exposure). All treated groups were contrasted against untreated groups, which was considered as a control group and built on the results from cellular uptake training, both RhB-loaded DOX-loaded NPs and blank NPs presented high cellular absorption in the early hours (around 100% uptake at first 0.5 h). High cellular absorption can be justified by the charge-shifting and size-shrunken potential of these engineered NPs^53^. This is due to the reduction cleavage of disulfide bonds due to the high level of GSH (10 mM) in the TME and changing of negative surface charges of COO^-^ groups of poly acrylic acid chain to less negative charge due to the protonation of these groups at slightly acidic pH of the tumor microenvironment^41^. Therefore, the ability of size shrinkage and charge-shifting is an advantage for in vitro improved MDA-MB-231 cellular uptake of the engineered pH/redox dual-responsive (PAA-b-PCL-S-S-PCL-b-PAA) NPs.

**Figure 5:**
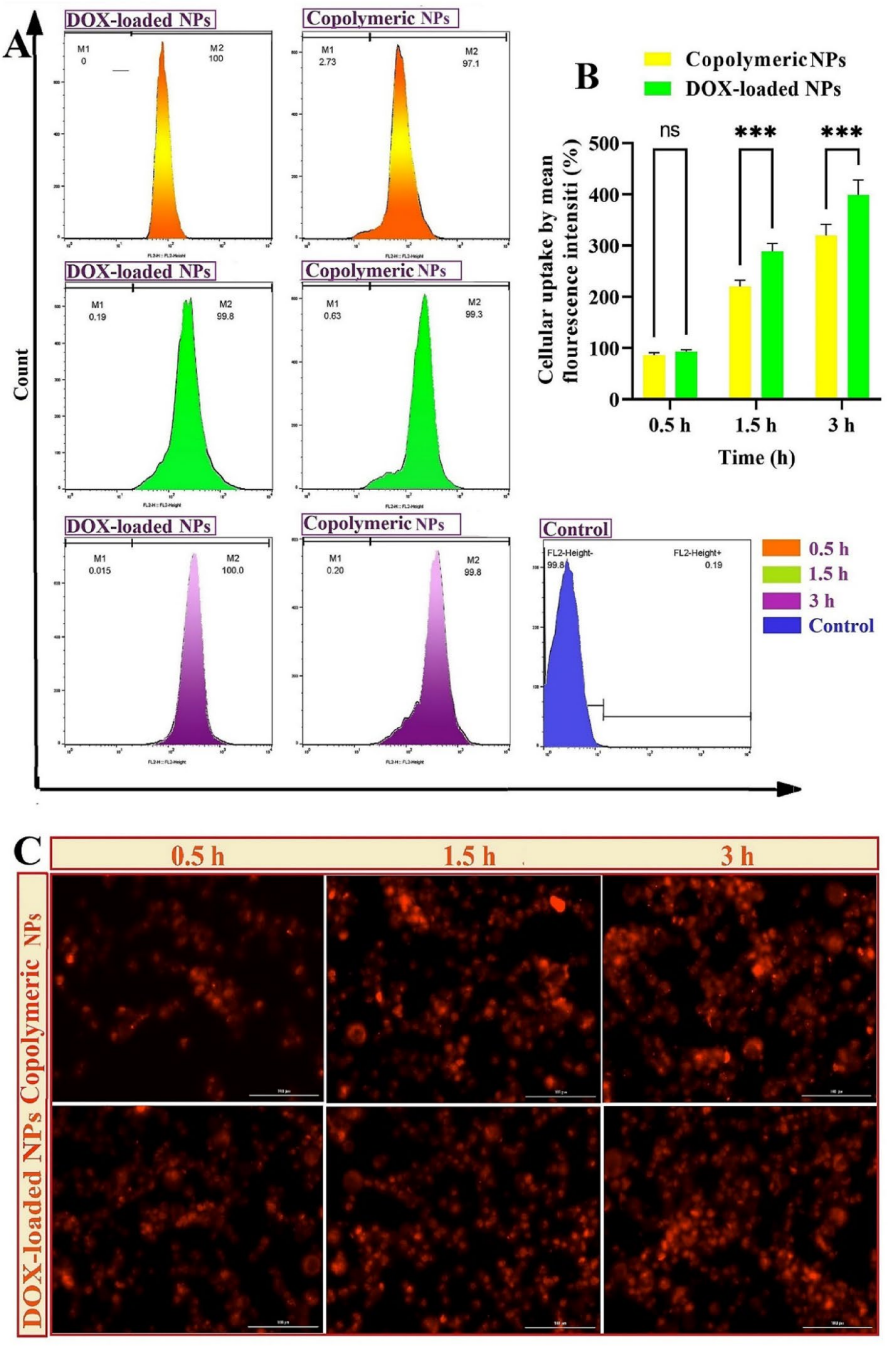
Outcomes of intracellular absorption of rhodamine B-labeled DOX-loaded (PAA-b-PCL-SS-PCL-b-PAA) NPs and rhodamine B-labeled (PAA-b-PCL-SS-PCL-b-PAA) NPs in various time intervals: 0.5, 1.5 and 3 h, at TME using flow cytometry (**A**); Graphs of mean fluorescence intensity (%) of intracellular uptake of RhB-labeled blank and DOX-loaded (PAA-b-PCL-SS-PCL-b-PAA) NPs in diverse time breaks: 0.5, 1.5 and 3 h, using flow cytometry; (diversities among treatments were statistically meaningful with the *p*-value ˂ 0.001) (**B**); Cytation 5 cell imaging multi-mode images of RhB-labeled blank and DOX-loaded (PAA-b-PCL-SS-PCL-b-PAA) NPs internalization at 0.5, 1.5 and 3 h. (All of them are treated by MDA-MB-231 cell lines) (**C**).

According to the cell internalization investigation at pH 6.5 (Fig. 5A), a high level of internalization (approximately 100% cell uptake) was obtained at all treatment times. To quantify the cellular uptake, the flow cytometry analysis of MDA-MB-231 treated with RhB-labelled NPs was assessed for 0.5, 1.5, and 3 h (Fig. 5A). The results demonstrated that following treatment of cells with the DOX-loaded NPs and blank NPs, mean fluorescence intensity (%) (MFI) was reported 92.5, 289, 399, and 86, 220, 320 over 0.5, 1.5, and 3 h, respectively. These outcomes demonstrated that copolymeric NPs uptake to MDA-MB-231cells was increased significantly (*p*-value ˂ 0.001) in a time-dependent manner. Referring to Fig. 5B, the MFI was gone up with time (0.5, 1.5, and 3 h). Likely, the mean fluorescence intensity of RhB-labeled drug-loaded NPs is much higher than RhB-labeled blank NPs (*p*-value < 0.001). RhB-labeled drug-loaded NPs have higher cellular uptake compared to RhB-labeled blank NPs because of the charged reversed from negative surface charge of blank NPs (− 17.8 mV) to positive at drug loaded NPs (+ 6.8 mV).

Similarly our results, Rahmani et al. depicted a lower cell uptake of PLGA-based blank NPs into MDA-MB-231 cell lines (33%, 60%, and 81% at 0.5, 1.5, and 3 h)^52^. The outcomes of cell internalization of rhodamine B-labeled DOX-loaded (PAA-b-PCL-SS-PCL-b-PAA) NPs and rhodamine B-labeled blank NPs achieved with fluorescent microscopy have proved the findings of the cell uptake obtained by flow cytometry (Fig. 5C).

As shown in Fig. S10A, comparison of the cellular uptake of blank NPs at pH 7.4 and 6.5, revealed that the cellular uptake of NPs at pH 6.5 is significantly higher than that at 7.4 (*P* < 0.001), indicating the effect of acid-triggered enhancement of cellular uptake at the.

tumor site. MFI of blank NPs at pH 6.5 and 7.4, was reported 86, 220, 320 and 43.6, 142, and 246 over 0.5, 1.5, and 3 h, respectively (Fig. S10B). The process of facilitating internalization of the nanoparticles can be described as follows: Initially, the acidic TME stimulates a decrease in the surface charge of the NPs, resulting in a less negatively surface charge of NPs. This reduced negative surface charge of NPs leads to a higher cellular uptake because of the weak electrostatic repulsion forces between NPs and negative charges of the cell membrane^54^. Furthermore, the elevated amount of GSH within tumoral tissues trigger the size shrinkage response in the NPs. Therefor, the rapid and abundant uptake rate of engineered NPs, substantiated the assertion that designed NPs can adjust the charge and size sensitivity in response to the TME stimuli for improved cell internalization.

### Apoptosis assays

#### The effect of DOX-loaded (PAA-b-PCL-SS-PCL-b-PAA) on cell cycle distribution in the breast cancer cell line

**Figure 6:**
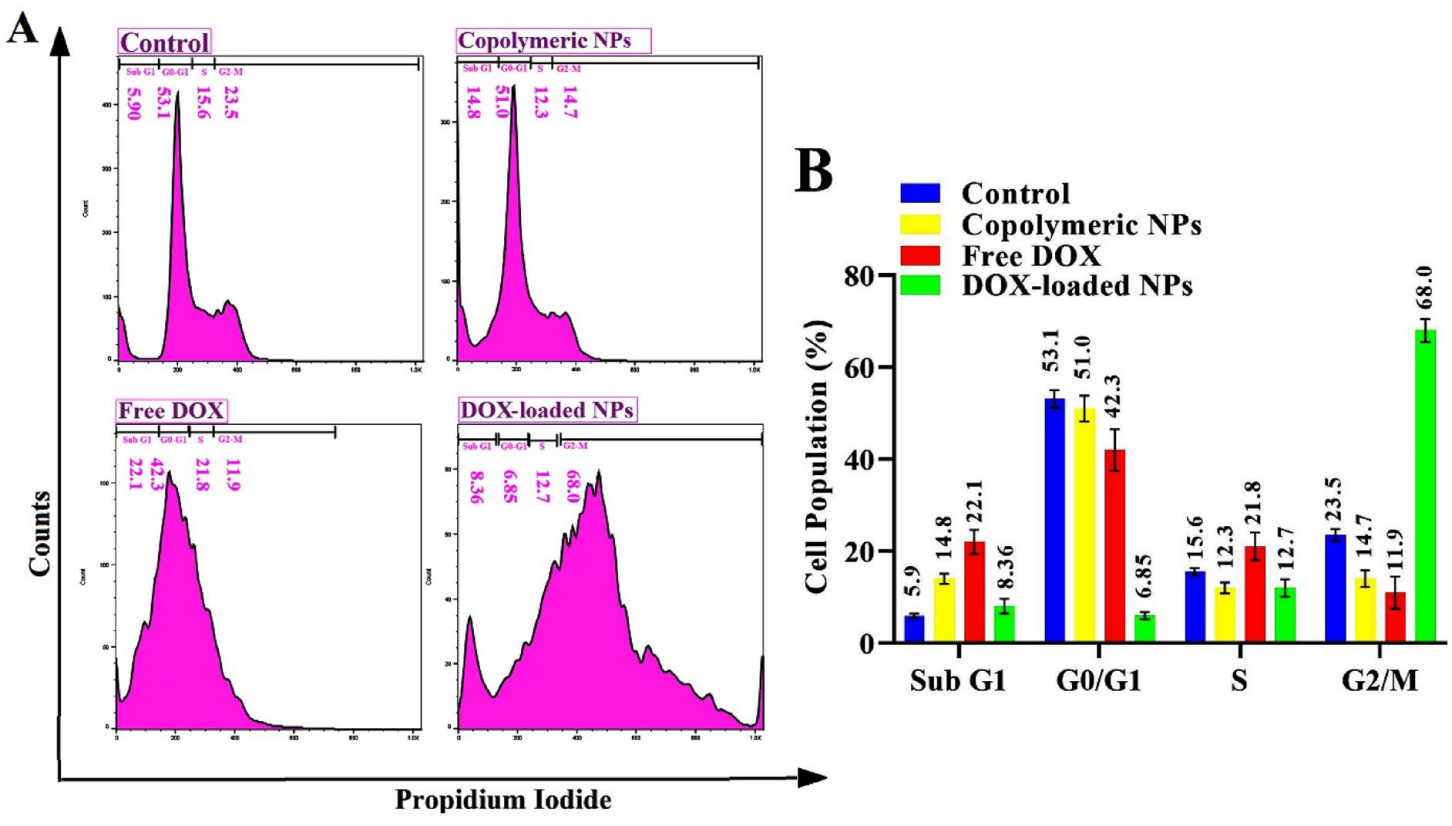
Histograms of cell cycle distribution results of MDA-MB-231 treated by free DOX, DOX-loaded NPs, polymeric NPs, and untreated cells as control group obtained by flow cytometry technique (**A**); and Quantitative plot of the cell population (%) profile in phases of the cell cycle (S, G2/M, G0/G1, and Sub G1) (**B**).

Cell cycle assays were conducted on MDA-MB-231 cancer cells treated via DOX-loaded (PAA-b-PCL-SS-PCL-b-PAA) NPs leading to a significant increase in G2/M phase cell population (68%), while free DOX treatment group lead to SubG1 (22%) and S (21%) arrest (Fig. 6). Similar to our results, MDA-MB-231 cells treated by DOX-loaded β-CDg-PMA-co-PLGA NPs showed G2/M and a lesser percentage of S arrest than the control group^54^. Poursalehi et al. reported that DOX-loaded-NPs lead to G2/M and SubG1 arrest in MCF-7 cells, while free DOX showed SubG1 arrest^55^. However, failing the G2-M arrest checkpoint may permit cells to enter mitosis. At the same time, DNA is damaged and subsequently induces the apoptosis pathway, and enhanced this effect may increase the cytotoxicity of chemotherapy. Inducing higher G2/M phase arrest has also been associated with enhanced inducing apoptosis. Like Annexin-V apoptosis assay results, both blank NPs and control groups showed almost the exact profile of cell cycle perturbance, therefore these developed copolymeric NPs showed no toxic effect on MDA-MB-231 cells. As shown in Fig. 5, around 53% and 51% of untreated cells (control group) and cells treated with blank NPs were in the G0-G1 phase. Pakravan et al. reported that free DOX, DOX-loaded HGNSs@Pol, and DOX-loaded GNCs@Pol treatment groups arrested MCF-7 cell cycle profiles in SubG1 and S phases which evidences DOX release from nano-formulations^56^.

#### Evaluation of apoptosis after treatment of MDA-MB-231 with DOX-loaded (PAA-b-PCL-SS-PCL-b-PAA) nanocarriers

**Figure 7:**
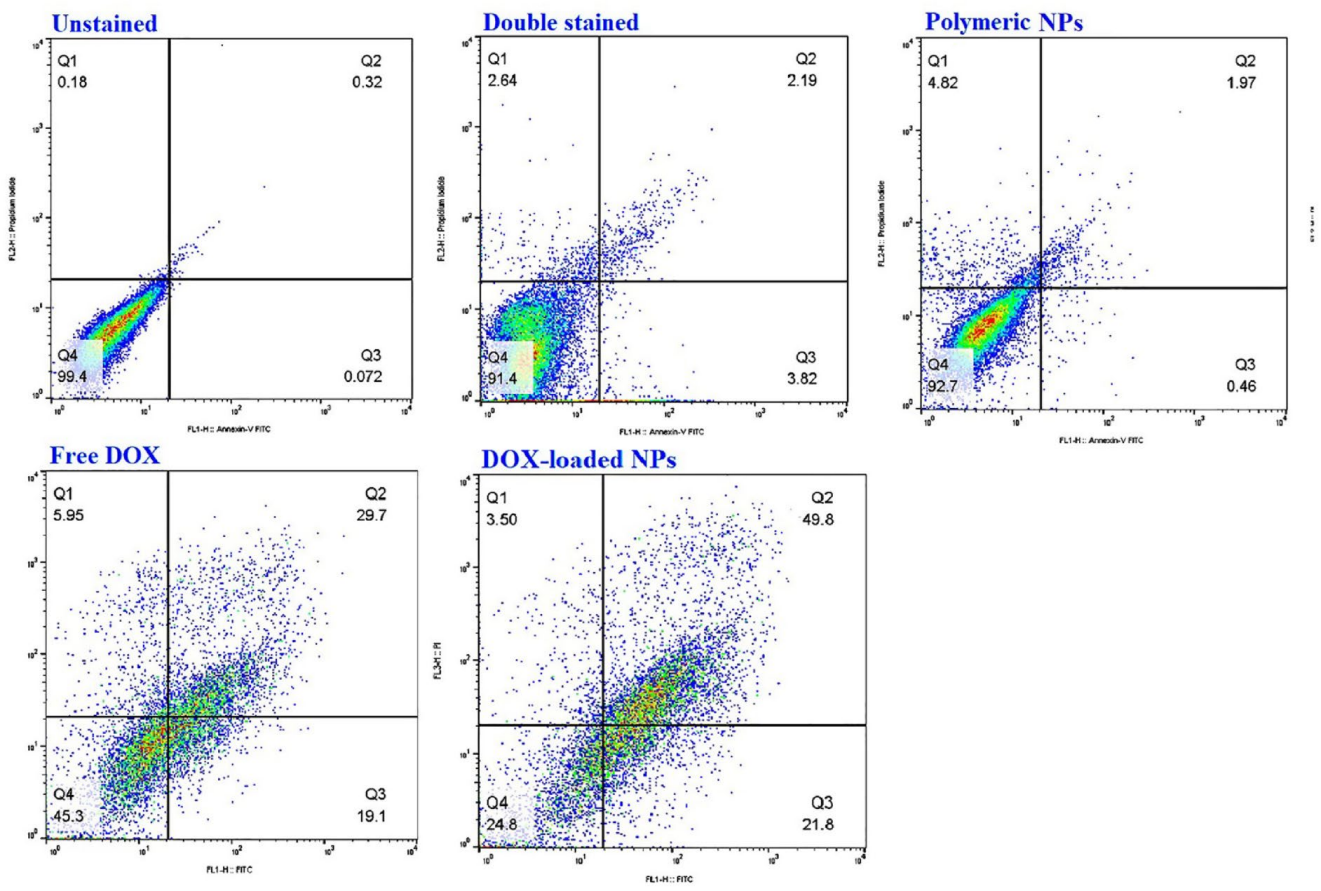
Apoptotic analysis via Annexin V-FITC/PI assay, resolution by flow cytometry, for MDA-MB-231 cells treated with blank (PAA-b-PCL-SS-PCL-b-PAA) NPs, free DOX and DOX-loaded (PAA-b-PCL-SS-PCL-b-PAA) NPs, unstained cells, and Annexin-V/propidium iodide double-stained cells were considered as control.

Annexin-V/FITC staining is the standard quantitative method, conducted through flow cytometry technique for the study of the effect of treatment on cell death via necrotic, early apoptotic, and late apoptotic cells. Quantitative outcomes of the Annexin V-FITC analysis are presented in Fig. 7. The viable cell population in the blank NPs group was near the control one. After the treatment of cancer cells with IC_50_ dose of DOX-loaded NPs and free DOX, the population of apoptotic cells was 71.6 and 49.8%, respectively. Our results demonstrated that loading of DOX in NPs could increase the percentage of apoptosis by 1.5-fold compared with DOX alone in MDA-MB-231 cells. Treating of breast cancer cells with DOX-loaded (PAA-b-PCL-SS-PCL-b-PAA) not only induced statistically significant total apoptosis, but also the most marked change was in a late phase of apoptosis compared to DOX alone treatment (49.8% versus 29.7%). Therefore, assuredly the percentage of apoptotic cell proportion in the DOX-loaded NPs medicated group had enhanced significantly, in contrast to other groups. Comparison of the higher apoptotic ratio of nano-formulations compared to free DOX revealed their higher cellular uptake and hence higher drug release inside targeted cells.

Similar results were observed in a study by Sabzi et al. in which the apoptotic percentage of MDA-MB-231 treated with free DOX was 47%, while DOX-loaded PCL-co-P(MA-g-CA) NPs was increased to 58%^57^.

#### The gene expression level of pro and anti-apoptotic genes over treatment of MDA-MB-231 cells with DOX-loaded (PAA-b-PCL-SS-PCL-b-PAA)

The mechanism of apoptosis in the treated MDA-MB-231 cells was evaluated to survey the essential role of the caspase-dependent programmed cell death routes in cell division to prevent uncontrolled cancer and balance cell growth.

**Figure 8:**
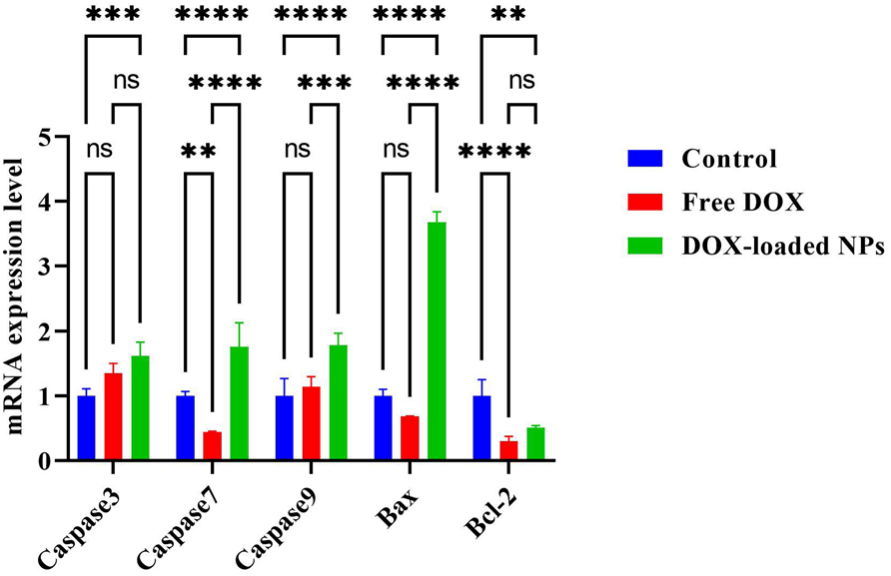
QRT-PCR results of apoptotic effect of free DOX and DOX-loaded NPs treated with MDA-MB-231 cells. Untreated cells are considered controls.

Bcl-2 as the anti-apoptotic protein and Bax as the pro-apoptotic protein is controlled the mitochondrial release of cytochrome-c. Therefore, the effect of DOX-loaded copolymeric NPs on the gene expression level of Bcl-2, Bax, and caspases 3, 7, and 9 were assessed using QRT-PCR. Results showed a noticeable increase in the gene level of Bax, caspases 3, 7, and 9, and a decrease in the expression of Bcl-2 following the treatment of cells with DOX-loaded nanoformulations in comparison with the DOX alone and control groups (Fig. 8). Cytochrome c release from injured mitochondria can activate caspase 9 as an upstream initiating caspase, thus goes on to cleave caspases 3 and 7, initiating the caspase cascade as they cleave several other cellular targets. This procedure is a signal of the internal apoptosis pathway. Since Bcl-2 inhibits apoptosis, a low degree of Bcl-2 mRNA was under apoptosis. Expression of apoptotic genes, which was notably significant in MDA-MB-231 cell lines treated with the DOX-loaded copolymeric NPs, was confirmed by the results of the Annexin-V assays and cell cycle arrest tests. The RT–PCR apoptosis–focused gene outcomes demonstrated that the intrinsic apoptosis pathway could benefit from the expression pattern through the caspase 7/Bcl-2, caspase 9, and caspase 3/Bax axis. Rahmani et al. reported a higher stratum of caspase-dependent apoptosis and intrinsic apoptosis root in the MDA-MB-231 cell line was induced by DOX-loaded β-CD-g-PMA-coPLGA NPs^54^. Sabzi and coworkers presented that DOX-loaded (PCL-co-P(MA-g-CA)) NPs induced apoptosis to breast cancer cells through caspases 3, 7, and 9 and Bax/Bcl-2 intrinsic route^57^. Our findings agreed with other studies, which found a higher amount of cell death after nanoformulation therapy of cancer cells.

#### Investigation of proteins involved in apoptotic signaling by western blotting

**Figure 9:**
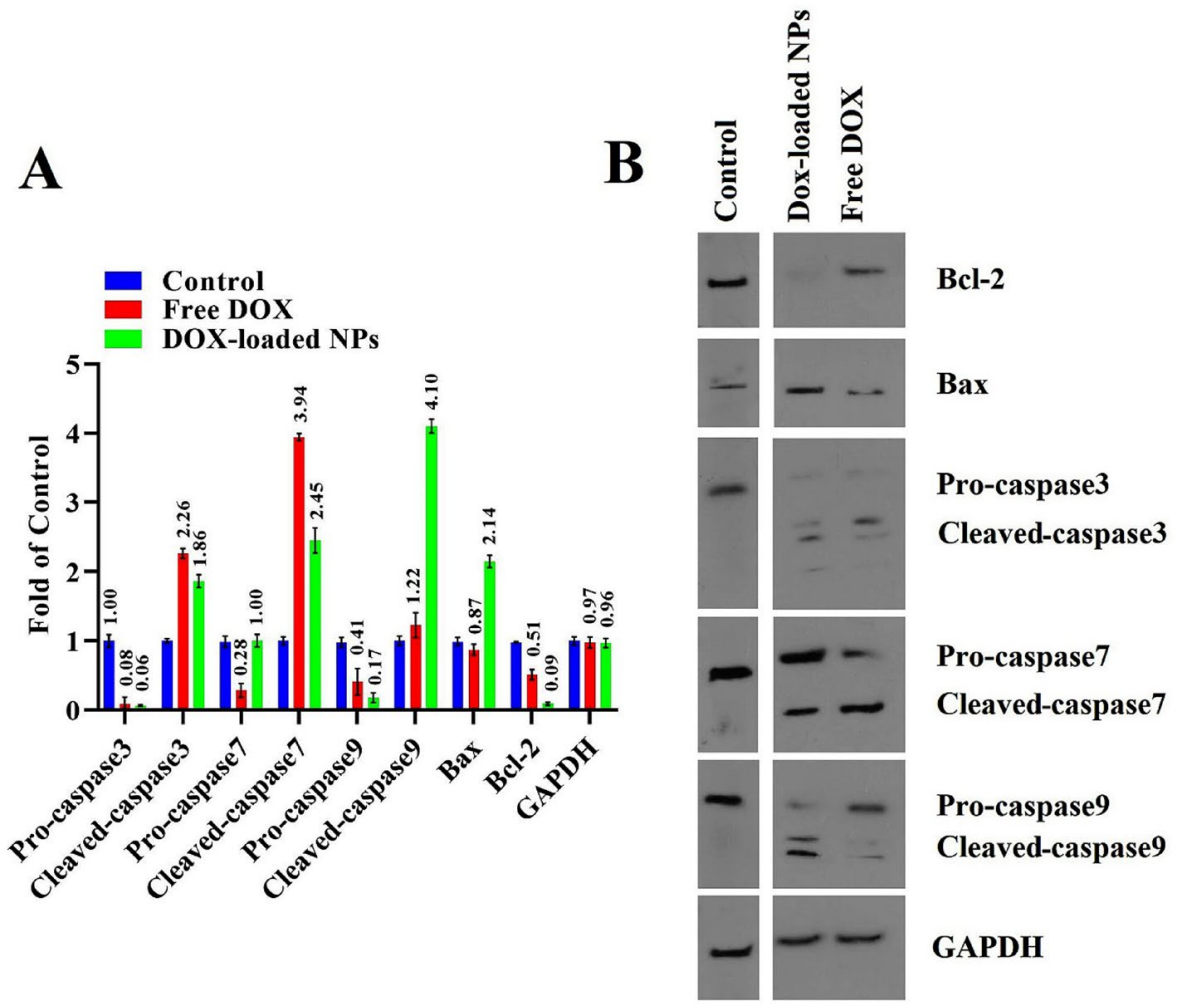
Plots of protein mood changes linked to the control group (protein expression = 1) provided from western blotting (MDA-MB-231 were treated with the concentration of IC_50_ dose of DOX-loaded NPs and free DOX) (**A**), and western blotting images of the MDA-MB-231 cells treated with DOX-loaded NPs and DOX (IC_50_ dose) (**B**). Untreated cells were considered the control group.

Western blotting would figure out the effect of DOX-loaded (PAA-b-PCL-SS-PCL-b-PAA) NPs on the protein level of Bax, Bcl-2, pro-caspases 3, 7, and 9, and cleaved caspase 3, 7 and 9 because it was adduced by real-time PCR analysis that the topmost level of innate caspase-dependent gene level apoptosis root was increased by DOX loaded-NPs. Figure 9 presented the western blotting outcomes and quantitative comparison of protein expression in MDA-MB-231 medicated by DOX-loaded NPs and free DOX. Our results showed a noticeable increase in the protein level of cleaved-caspase 9 (3.36-fold), and Bax (2.46-fold), and decline in expression of pro-caspase 3 (0.75-fold), pro-caspase 9 (0.41-fold), and Bcl-2 (0.17-fold), concerning the free DOX. Increasing the Bax expression ratio to Bcl-2 led to the release of mitochondrial proapoptotic proteins cytochrome c, which formed a large complex with pro-caspase 9 and Apaf-1. This structure creates the apoptosome with the expression of pro-caspase-9. Upregulation brought about cleavage of caspase 9 aligned with pro-caspase 9 downregulation. Increased adjustment of cleaved-caspase 9 leads to pro-caspases 7 and 3 upregulation and their cleavage (upregulation of cleaved-caspases 7 and 3). As a final point, upregulation of cleaved-caspases 7 and 3 resulted in increased rates of death substrate and DNA damage/fragmentation. Original western blot images were presented in Fig. S11. Therefore, along with the RT-PCR outcomes, western blotting revealed that DOX-loaded NPs induced apoptosis in MDA-MB-231 through innate mitochondrial root because of a noticeable boost in the expression of cleaved-caspases 3, 7, and 9. In a study by, Li et al., free DOX-induced apoptosis in MDA-MB-231, with the regulation of Bax/Bcl-2, caspase 3, and PARP signaling pathways, therefore DOX decreased the levels of anti-apoptotic Bcl-2 marker and increased the levels of pro-apoptotic marker Bax, cleaved PARP, and cleaved caspase 3^58^.

Western blotting results revealed that the treatment of MDA-MB-231 cells by DOX-loaded (PAA-b-PCL-S-S-PCL-b-PAA) nanoparticles could induce apoptosis via activation of the (caspase-dependent) intrinsic mitochondrial apoptosis route as proven by the gene level of RT-PCR assay.

## Conclusion

A novel TME-responsive DOX-loaded nano drug delivery system (NDDS) with pH/redox dual-responsive ability to combat MDA-MB-231 tumors was successfully developed in this study. To achieve this concept, a well-engineered (PAA-b-PCL-S-S-PCL-b-PAA) copolymeric NPs with size shrinkage and charge-shifting ability after exposure to the tumor extracellular microenvironment was synthesized, which induced multiple advantages, including smartly controlled dual pH/redox -triggered intracellular DOX release pattern, increased tumor cell uptake and site-specific targeted doxorubicin delivery and improved MDA-MB-231 tumor cells death via apoptosis.

According to i*n vitro* cytotoxicity assay tests, DOX-loaded NPs showed higher cytotoxicity (IC_50_ = 0.386 µg·mL^−1^) than free DOX (IC_50_ = 0.607 µg·mL^−1^) against MDA-MB-231 cancer cells. Cell uptake results showed that were internalized quickly (0.5 h) and completely (100%) into MDA-MB-231, because of their favorable size and zeta potential. The results of cell cycle arrest and apoptosis evaluation by Annexin V revealed that DOX-loaded NPs caused an intense disruption in the cell cycle pattern (60% G2-M arrest) with exceptional apoptosis induction (more than 70%) compared to the free DOX (49.8%). The results of western blotting (at protein level), and RT-PCR (at gene level) assays showed that DOX-loaded NPs induced apoptosis in MDA-MB-231 breast cancer cells via Bcl-2/Bax, caspases 3, 7, and 9 dependent inherent mitochondrial apoptosis routes. In conclusion, the novel DOX-loaded pH/redox dual-responsive (PAA-b-PCL-SS-PCL-b-PAA) copolymeric nanodrug delivery systems (NDDSs) has led to the design of charge-shifting and size shrinkage concepts with minimum side effect outstanding anticancer drug delivery system, which could be identified in vivo in similar forthcoming situations.

### Supplementary Information

Supplementary Information.

## Data Availability

All data generated or analyzed during this study are included in this published article [and its supplementary information files].
