# Supplementary material for: pH/redox responsive size‐switchable intelligent nanovehicle for tumor microenvironment targeted DOX release
Source: Sci Rep. 2023 Dec 18;13:22475. doi: 10.1038/s41598-023-49446-x (PMC10728153; doi:10.1038/s41598-023-49446-x)
Supplement: Supplementary file 1 — Supplementary Information. [file 41598_2023_49446_MOESM1_ESM.docx]

**Supplementary Information:**

# **pH/redox responsive size‐switchable intelligent nanovehicle for tumor microenvironment targeted DOX release**

Fahimeh Badparvar^1^, Ahmad Poursattar Marjani^1,^*, Roya Salehi^2,^*, and Fatemeh Ramezani^3^

*^1^Department of Organic Chemistry, Faculty of Chemistry, Urmia University, Urmia, Iran*

*^2^Drug Applied Research Center and Department of Medical Nanotechnology, Faculty of Advanced Medical Sciences, Tabriz University of Medical Sciences, Tabriz, Iran*

*^3^Department of Medical Nanotechnology, School of Advanced Medical Sciences, Tabriz*

*University of Medical Sciences, Tabriz, Iran*

* E-mail: [a.poursattar@urmia.ac.ir](mailto:a.poursattar@urmia.ac.ir); [salehiro@tbzmed.ac.ir](mailto:salehiro@tbzmed.ac.ir)


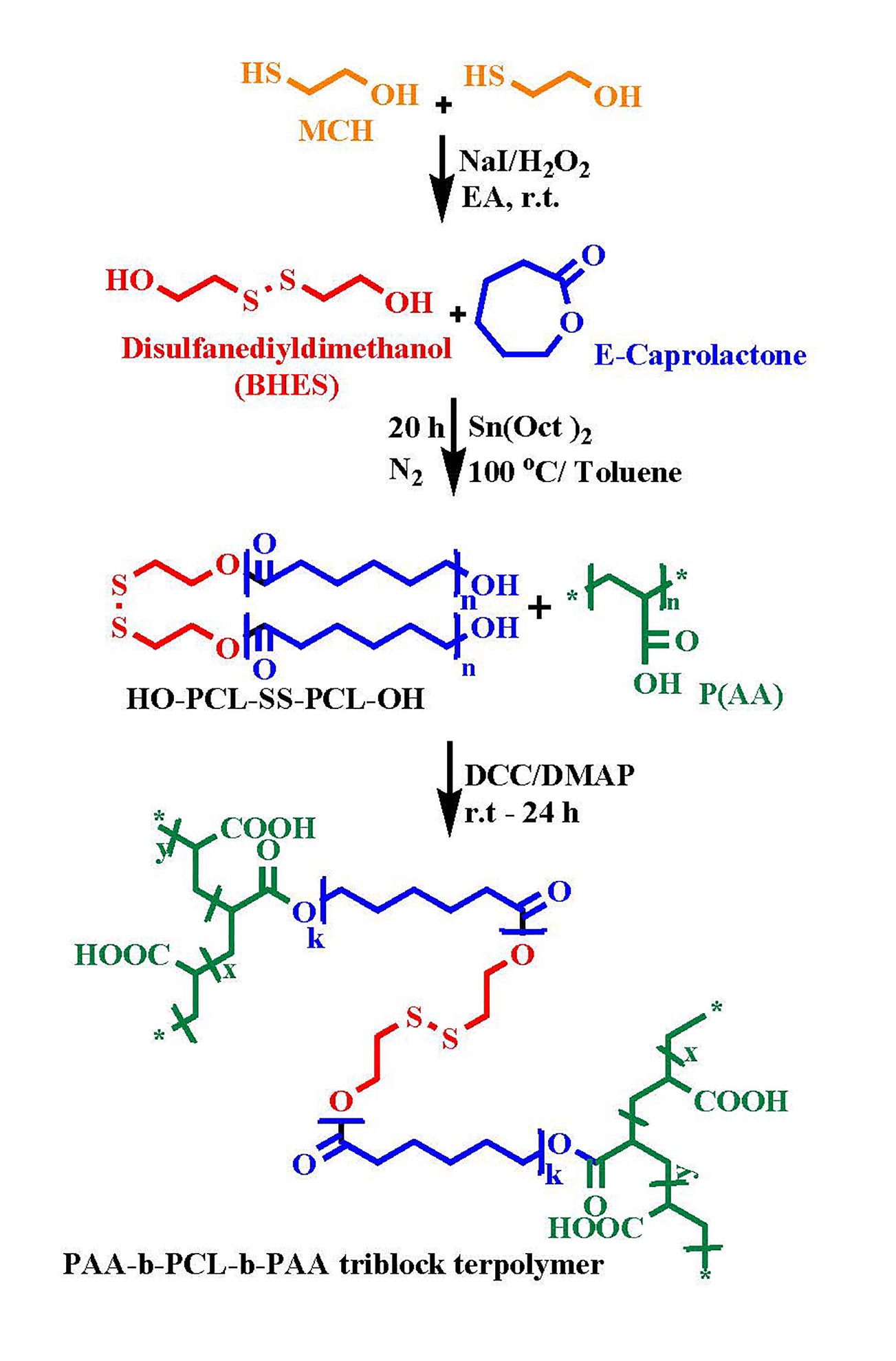


**Figure S1.** Schematic illustration of synthesis root of PAA-b-PCL-S-S-PCL-b-PAA triblock copolymer.

**Table S1**. Primers Sequence

| **Gene** | **Reverse primer (5′-3′)** | **Forward primer (5′-3′)** |
| --- | --- | --- |
| CASP-3 | CTACAACGATCCCCTCTGAAAAA | GAAATTGTGGAATTGATGCGTGA |
| CASP-7 | CACTGGGATCTTGTATCGAGGA | AGGGACCGAGCTTGATGATG |
| CASP-9 | GCACCACTGGGGTAAGGTTT | CTTCGTTTCTGCGAACTAACAGG |
| Bax | CAGCCCATGATGGTTCTGAT | TTCTGACGGCAACTTCAACT |
| Bcl-2 | GGCAACGATCCCATCAATCT | GGGAATCGATCTGGAAATCCTC |
| GAPDH | GCCATCACGCCACAGTTTC | ACAACTTTGGTATCGTGGAAGG |

**Characterization**

**Infrared spectroscopy study.** The chemical structure of synthetic intermediate molecules and copolymers can further confirm the FT-IR spectrum (Fig. S2).

To obtain information about the structures of the HS-CH_2_CH_2_-OH (Fig. S2A) HO-(CH_2_)_2_-SS-(CH_2_)_2_-OH (Fig. S2B) HO-PCL-SS-PCL-OH (Fig. S2C), PAA (Fig. S2D), and (PAA-b-PCL-S-S-PCL-b-PAA) (Fig. S2E), FT-IR spectroscopy was carried out.

According to Figure S2A, the absorption band in the broad frequency range 3201–3501 cm^-1^ corresponds to the hydroxyl of 2-mercaptoethanol. The band between 2880–2950 cm^-1^ is relevant to aliphatic C–H stretching. Also, –SH stretching of the 2-mercaptoethanol appears at 2562 cm^-1^, and the C–S bending appears at 938 cm^-1^, respectively.

Figure S2B shows the FT-IR spectrum of the BHES. Two apparent differences in observed bands with pure 2-mercaptoethanol suggest that oxidation of 2-mercaptoethanol was done. The first difference in the disappearance of peak frequency at 2561 cm^-1^ is related to the –SH group. Moreover, the addition of the peak at 592 cm^-1^, can correspond to the establishment of the S-S band.

In the case of Figure S2C, the chemical structure of the HOPCL-S-S-PCLOH copolymer was investigated by FT-IR spectroscopy. The peak at 1727 cm^-1^ is relevant to the tensile vibration of carbonyl groups. The intense 1187 cm^-1^ peak is associated with C–O–C. The absorption peak of the C–H bond, which introduces as a polymer backbone, has appeared at 2947–2867 cm^-1^. The peak observed at 3441 cm^-1^ is related to –OH groups of the PCL chain. The intense bands observed at 1244 and 1045 cm^-1^ correspond to the CO ester bonds involving the –OH groups of CO. At this stage, it is visible that the initiator spectrum has disappeared and has been replaced by the HOPCL-SS-PCLOH copolymer spectrum. This is due to the presence of numerous polymer chains in comparison to the initiator sections, which is indicated that the chain growth is successful.

As shown in Fig. S2D related to polyacrylic acid nanogel, a broad band at 3433 cm^-1^ corresponds to asymmetric stretching vibrations of the hydroxyl group. The peak at 2933 cm^-1^ is related to the –CH_2_ stretching mode of PAA hydrogel.

Furthermore, the prominent peak at around 1731 cm^-1^ is related to the stretching mode of the C=O group of the –CO_2_H group on the PAA chain, and CO_2_^-^ stretching vibrations bonds are seen at the 1445 cm^-1^ regions, respectively.

Figure S2E illustrates the FT-IR spectrum acquired for PAA-b-PCL-S-S-PCL-b-PAA, which comparing the synthesis steps, provides strong evidence for the correct formation of the PAA-b-PCL-S-S-PCL-b-PAA final copolymer so that all the indicator previous stage peaks related to HO-PCL-SS-PCL-OH and PAA hydrogel are visible in the last PAA-b-PCL-S-S-PCL-b-PAA copolymer spectrum. A significant signal occurs with the appearance of a Broad band of OH stretching vibrations peak at 3440 cm^-1^, which is weaker than the product of the previous stage and is due to the loss of some OH groups by the esterification method. Also, a double peak at 2948 and 2867 cm^-1^ is assigned to the symmetric and asymmetric –CH group of the PCL backbone. Another visible symptom is the shifting of the strong shoulder of C=O from 1727 to 1728 cm^-1^ due to relative contribution changes of two overlapped bands.


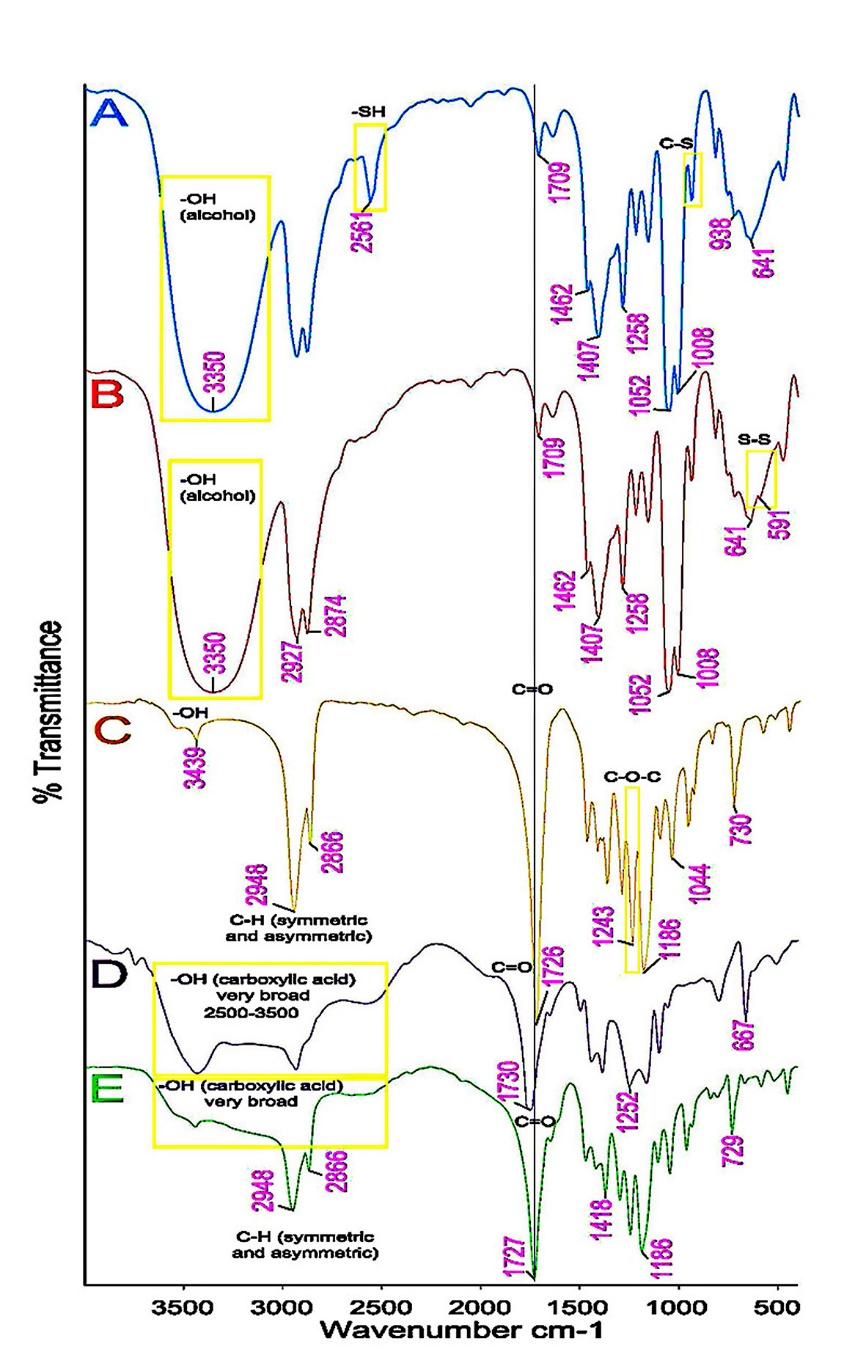


**Figure S2.** FTIR spectra of HO-CH_2_CH_2_-SH (**A**), HO-CH_2_CH_2_-SS-CH_2_CH_2_-OH (**B**), HO-PCL-SS-PCL-OH (**C**), PAA (**D**), and (PAA-b-PCL-S-S-PCL-b-PAA) (**E**)

**2.3. GPC analysis**

The molecular weight of the synthesized bi-functional HO-PCL-SS-PCL-OH polymer

was specified by GPC using tetrahydrofuran as eluent (1 mL/min). Figure S3 showed that it has Mw = 602663e3 g.mol^-1^ with PDI = 1.34 and a uniformly distributed polymer and indicated that the ring-opening polymerization reaction occurs. Then the high molecular weight graft polymers are obtained


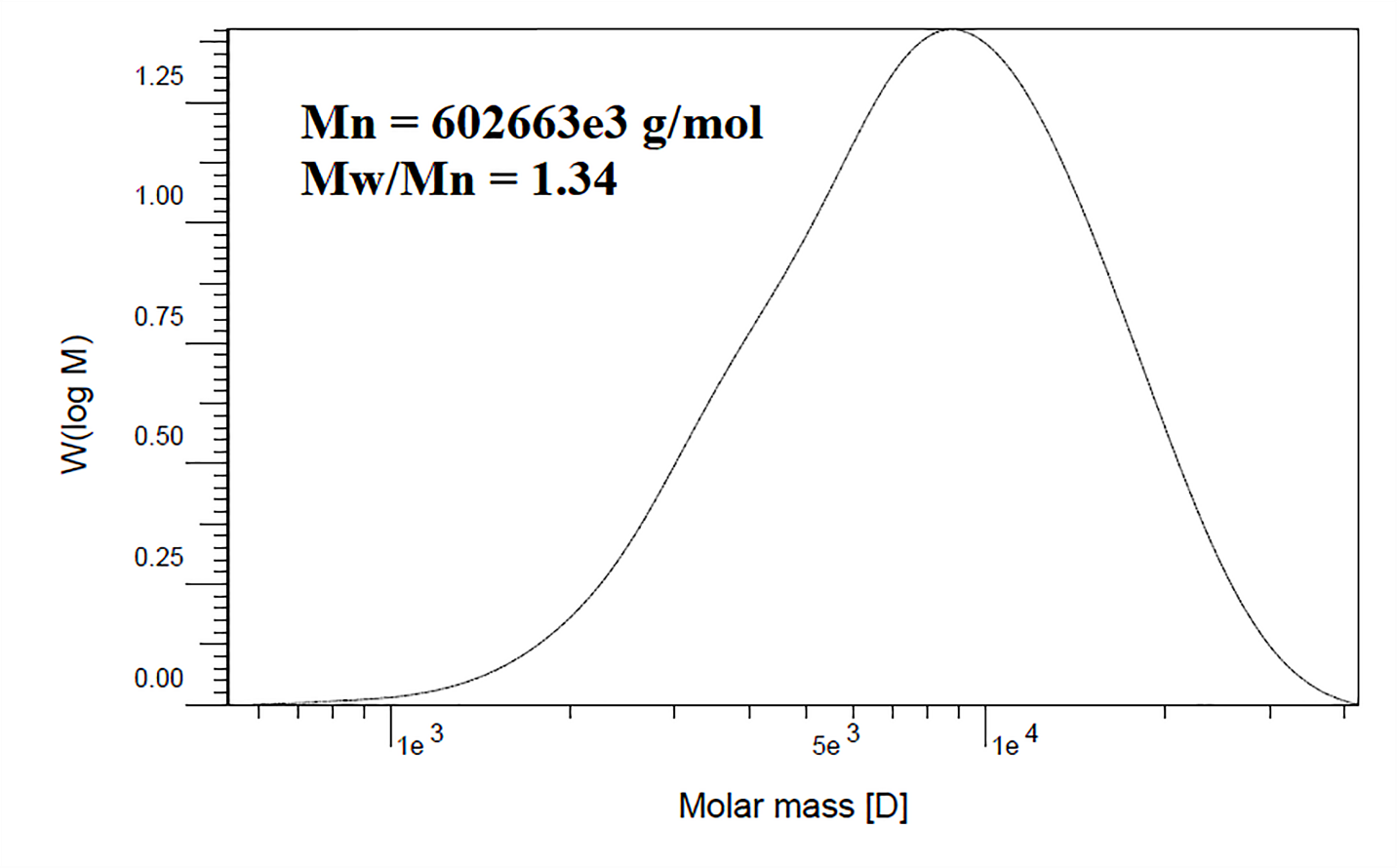


**Figure S3.** Molecular weights of synthesized HO-PCL-SS-PCL-OH


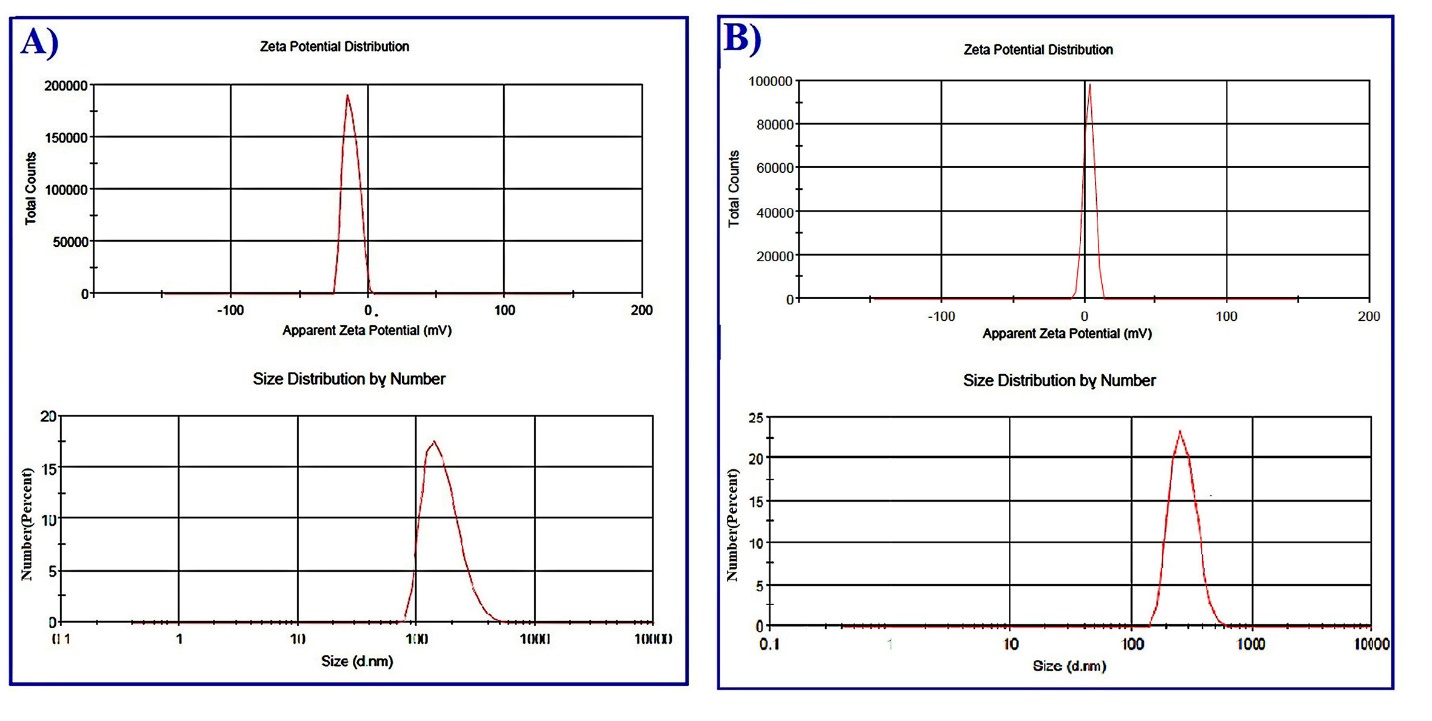


**Figure S4.** The zeta potential and average size of free NPs and (**A)** and drug-loaded NPs **(B)**.


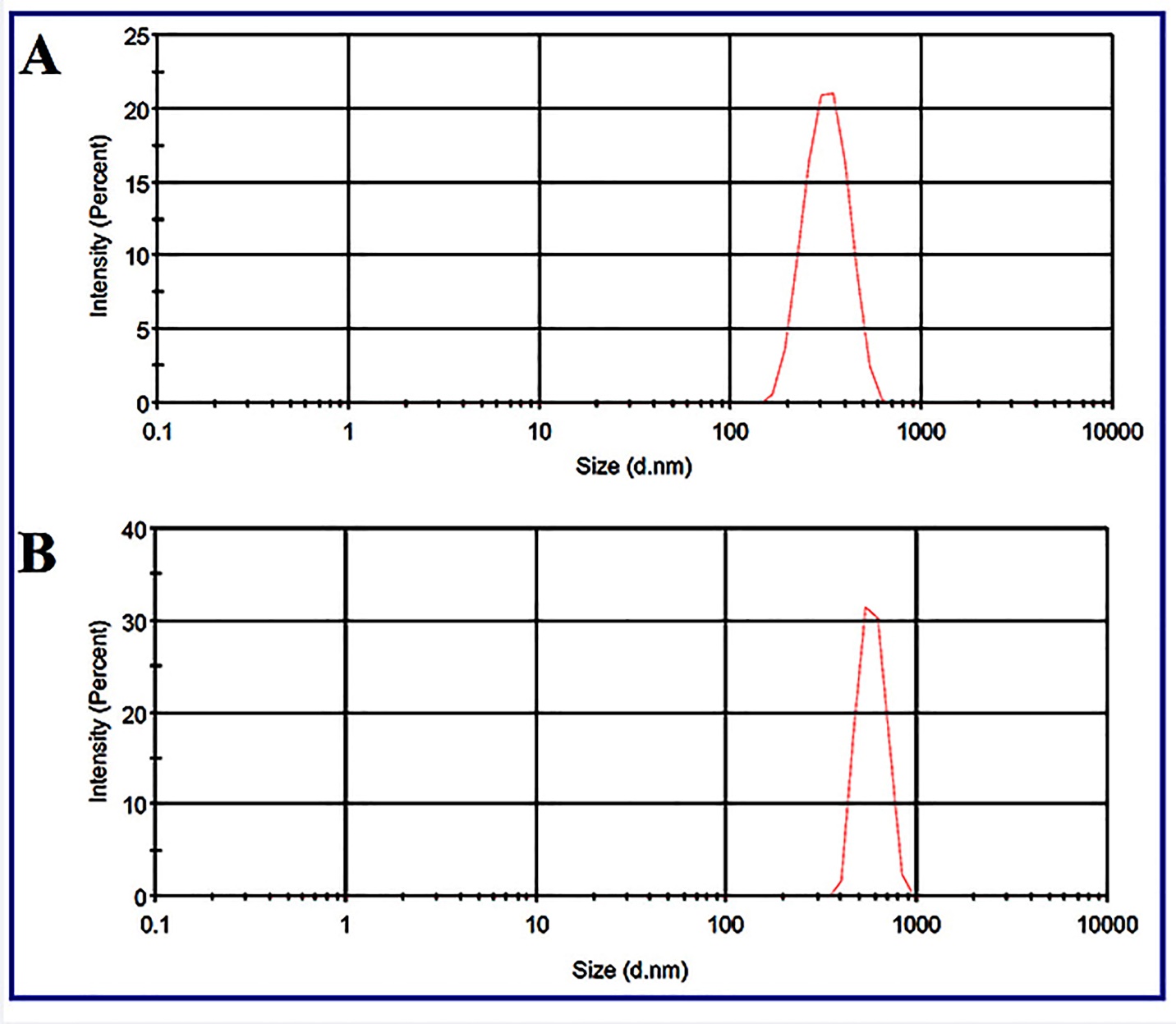


**Figure S5.** DLS results of (PAA-b-PCL-S-S-PCL-b-PAA) NPs after 2 mounts in PBS buffer (**A**) and distilled water (**B).**


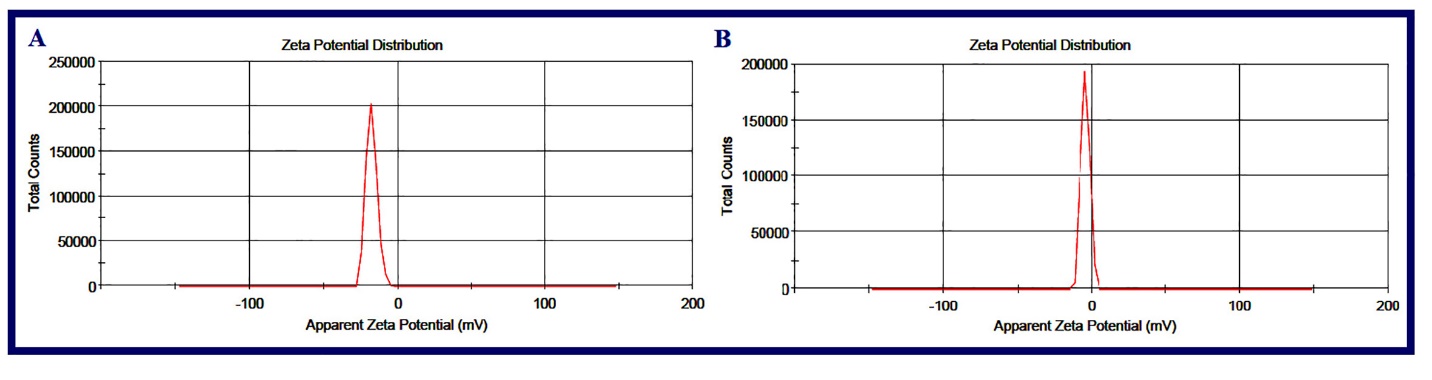


**Figure S6.** Zeta potential of (PAA-b-PCL-S-S-PCL-b-PAA) NPs after incubation (4 h) at pH = 7.4 (**A**) and pH = 6.5 (**B**)


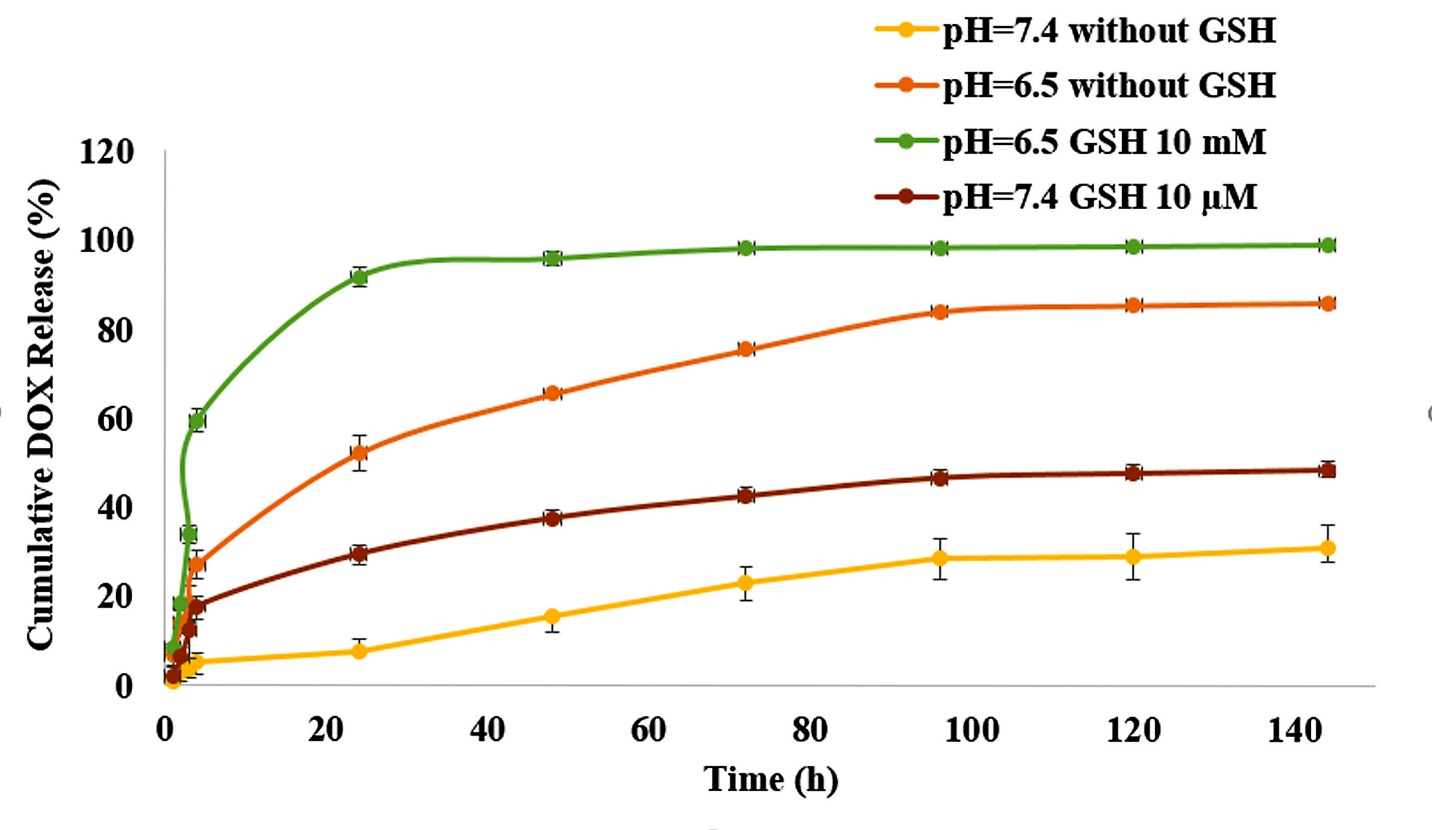


**Figure S7.** *In vitro* dual pH/redox triggered DOX release from PAA-b-PCL-S-S-PCL-b-PAA NPs in the absence of GSH and pH = 7.4 (yellow), 10 µM GSH and pH = 7.4 (brown), in the absence of GSH and pH = 6.5 (orange), and 10 mM GSH and pH = 6.5 green)

**
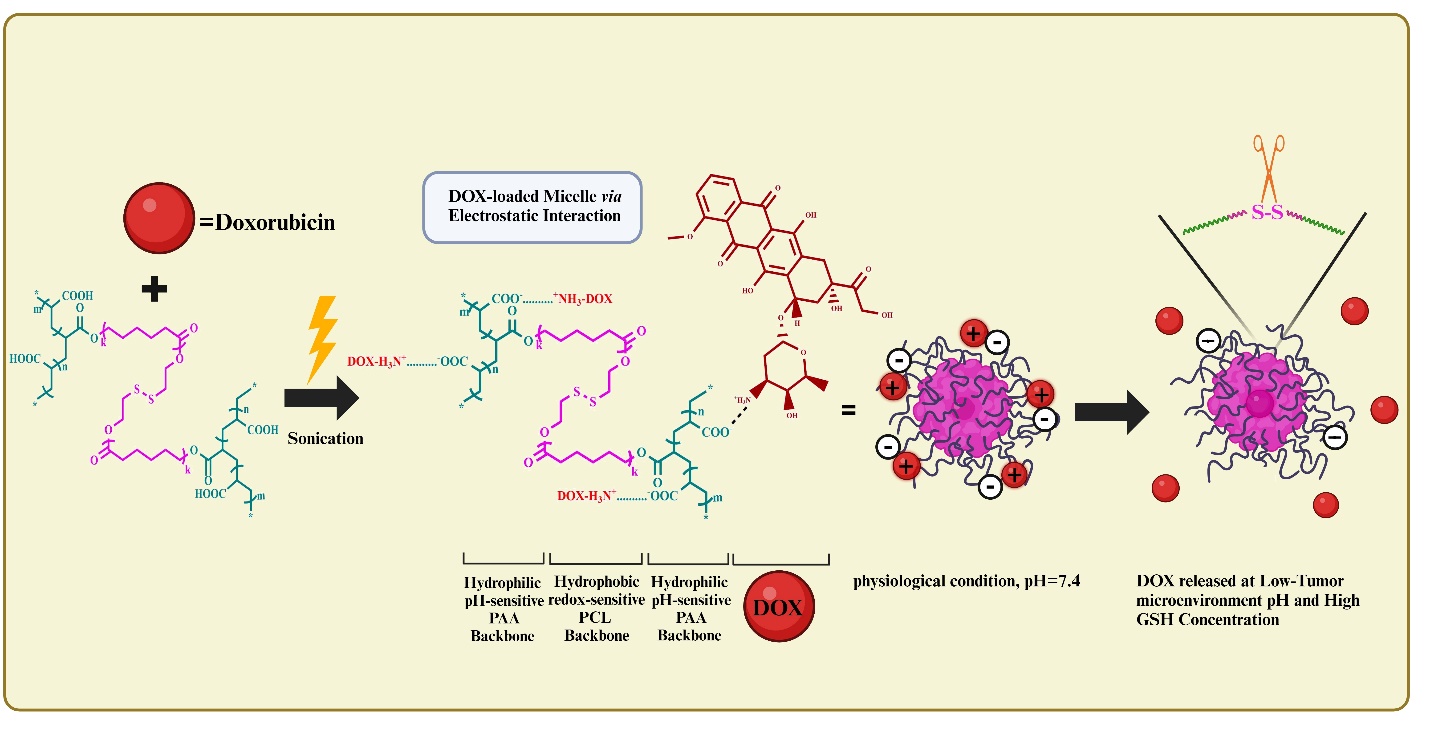
Figure S8.** The synthesizes of DOX-loaded pH/redox dual-responsive nanoparticles, DOX as a cationic hydrophilic drug, was encapsulated into the PAA-b-PCL-S-S-PCL-b-PAA copolymeric NPs *via* electrostatic interaction. The NPs were squeezed and deteriorated, which stimulated intracellular drug release. Also, at low pH, protonation of CO_2_^-^ groups of copolymeric NPs leads to the elimination of ionic interaction between NH_3_^+^ functional groups of DOX and NPs structure and further pH/redox triggered-release of DOX. This picture created by Biorender and has publication license.

**
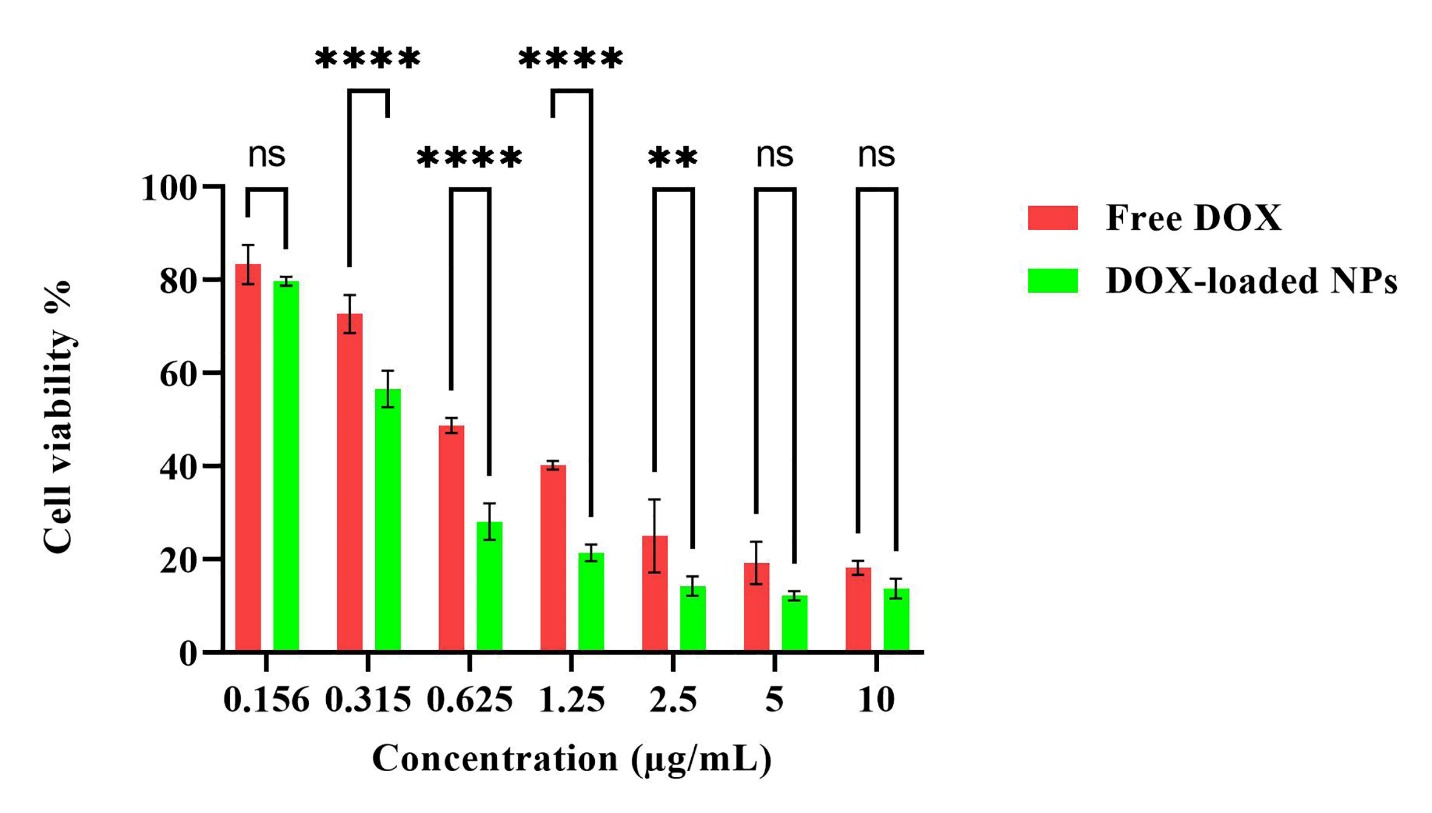
**

**Figure S9.** Viability (%) of MDA-MB-231 treated with free DOX and DOX-loaded NPs with various concentrations: 0.156, 0.315, 0.625, 1.25, 2.5, 5, and 10 μg/mL (48 h), by MTT assess.


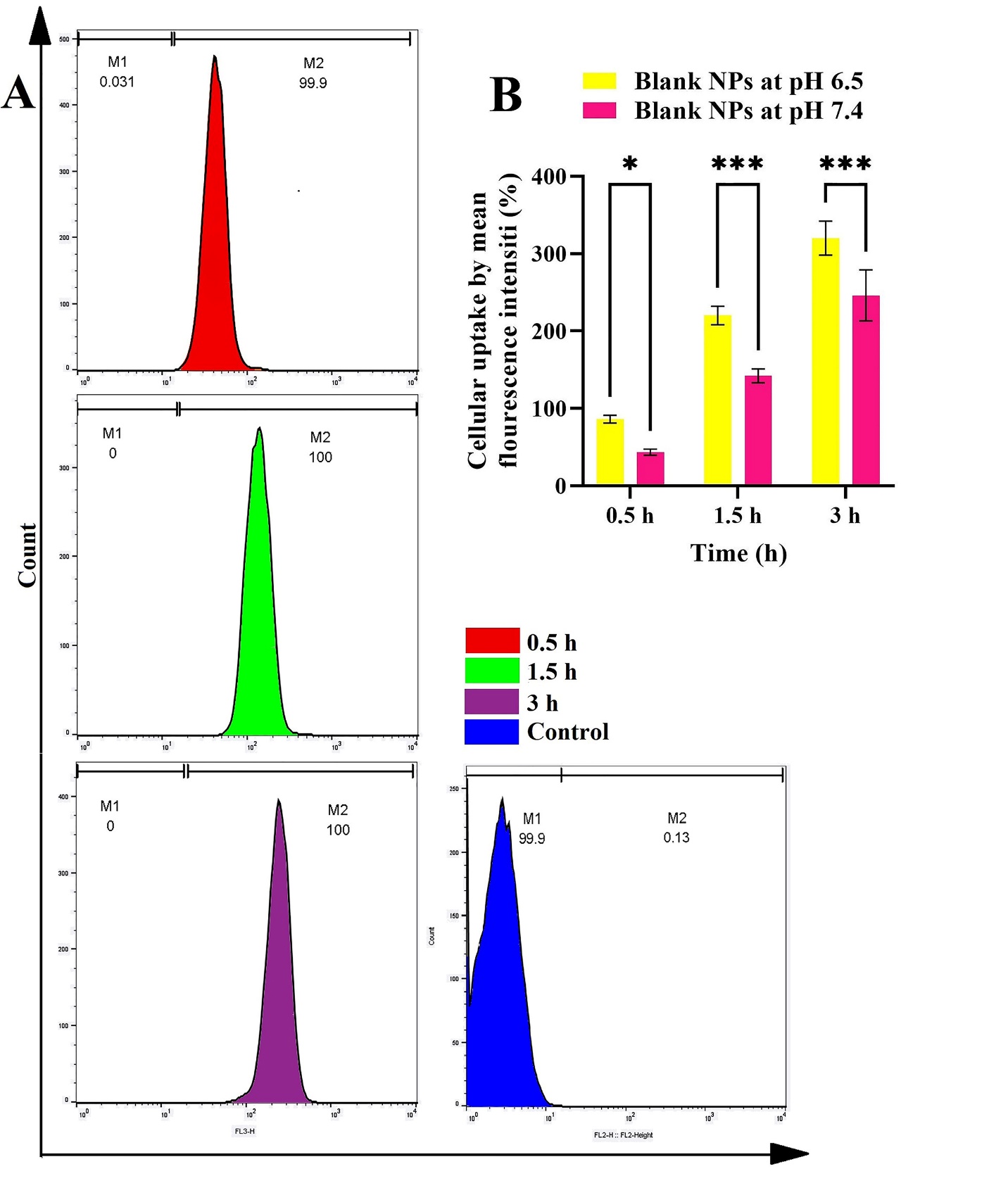


**Figure S10.** Uptake results of Rh-B-labeled blank (PAA-b-PCL-SS-PCL-b-PAA) NPs in various time intervals: 0.5, 1.5 and 3 h, at pH=6.5 using flow cytometry (**A**); comparison graphs of mean fluorescence intensity (%) of intracellular uptake of blank (PAA-b-PCL-SS-PCL-b-PAA) NPs at pH 7.4 and 6.5, using flow cytometry; (p-value ˂0.001) (**B**).

**2.4. Western blotting**

Western blot analysis was performed according mentioned protocol in the manuscript. All bands were obtained from same gel after some stripping and reprobing. Figure S11 showed the western blot images presenting GAPDH (**A**), pro. and cleaved caspase-7 (**B**), pro. and cleaved caspase-3 (**C**), pro. and cleaved caspase-9 (**D**), Bcl-2 (**E**), Bax (**F**) showed original. There are 3 bands in each original scanned image that were delineated in GAPDH image as follow: C = Control group, AAP = DOX-loaded (PAA-b-PCL-S-S-PCL-b-PAA) copolymeric nanocarrier, DOX = Doxorubicin (DOX). The images were cut on account of their lack of relevance to the subject matter under investigation.


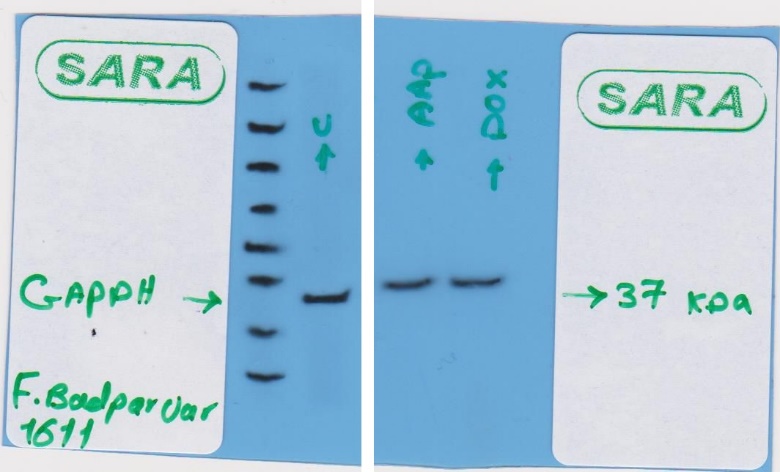


**Figure S11 (A).** Original western blot image of GAPDH. C = Control group, AAP = DOX-loaded (PAA-b-PCL-S-S-PCL-b-PAA) copolymeric nanocarrier, DOX = Doxorubicin (DOX).


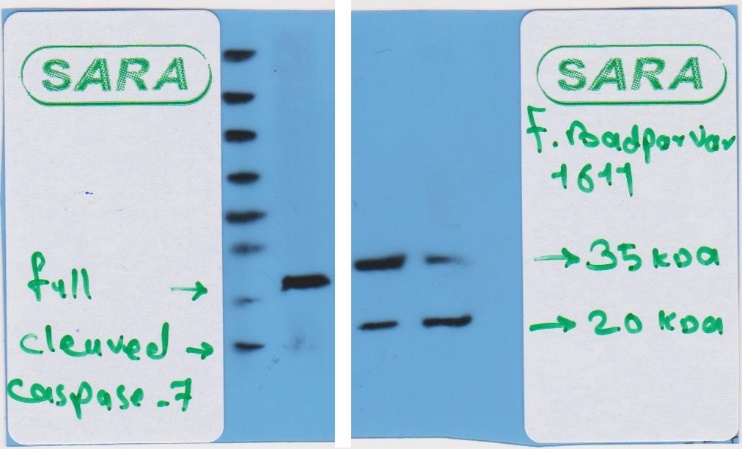


**Figure S11 (B).** Western blot original images for pro. and cleaved caspase-7. The label of “full” means is “pro. caspase-7”.


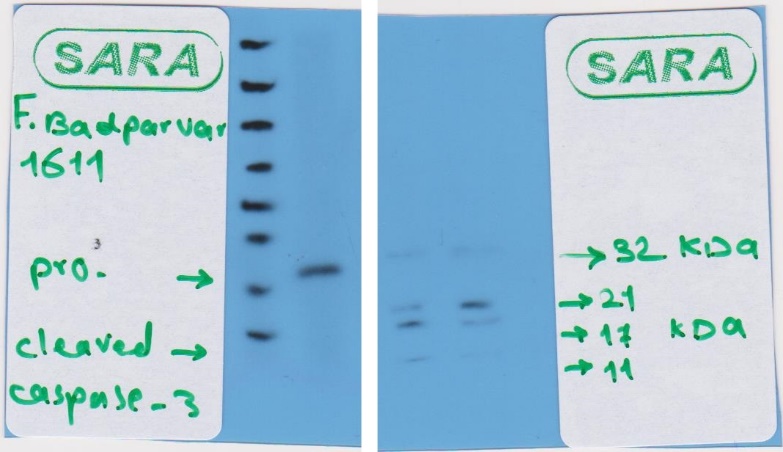


**Figure S11 (C).** Western blot original images for pro. and cleaved caspase-3.


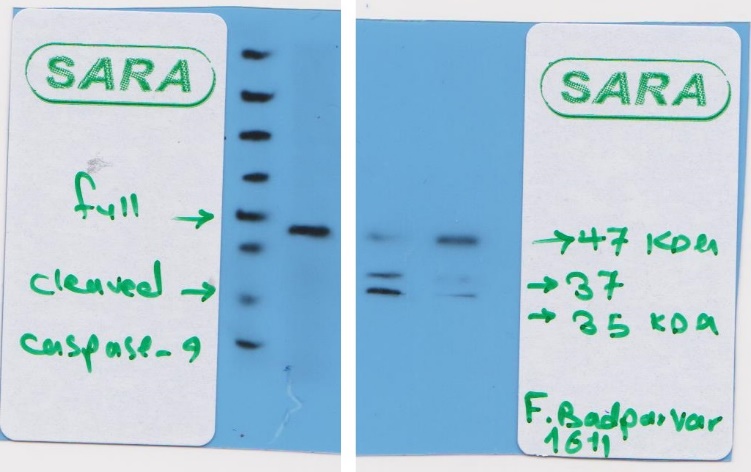


**Figure S11 (D).** Western blot original images for pro. and cleaved caspase-9. The label of “full” means “pro. caspase 9”.

**
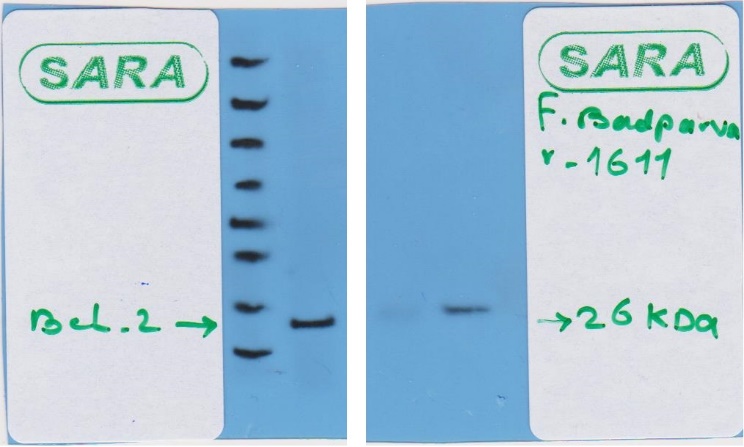
**

**Figure S11 (E).** Western blot original images for Bcl-2.


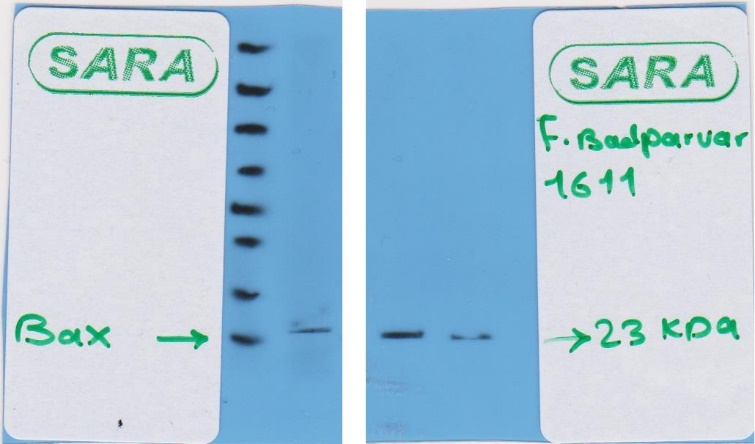


**Figure S11 (F).** Western blot original images for Bax.
